# Has *AlphaFold*3 achieved success for RNA?

**DOI:** 10.1107/S2059798325000592

**Published:** 2025-01-27

**Authors:** Clément Bernard, Guillaume Postic, Sahar Ghannay, Fariza Tahi

**Affiliations:** a Université Paris-Saclay, Université Evry, IBISC, 91020Evry-Courcouronnes, France; b LISN – CNRS/Université Paris-Saclay, 91400Orsay, France; Lawrence Berkeley National Laboratory, USA

**Keywords:** 3D RNA structure, *AlphaFold*3, deep learning, structure quality assessment

## Abstract

Predicting the 3D structure of RNA is a significant challenge despite ongoing advancements in the field. Although *AlphaFold* has successfully addressed this problem for proteins, RNA structure prediction raises difficulties due to the fundamental differences between proteins and RNA, which hinder its direct adaptation. The latest release of *AlphaFold*, *AlphaFold*3, has broadened its scope to include multiple different molecules such as DNA, ligands and RNA. While the *AlphaFold*3 article discussed the results for the last CASP-RNA data set, the scope of its performance and the limitations for RNA are unclear. In this article, we provide a comprehensive analysis of the performance of *AlphaFold*3 in the prediction of 3D structures of RNA. Through an extensive benchmark over five different test sets, we discuss the performance and limitations of *AlphaFold*3. We also compare its performance with ten existing state-of-the-art *ab initio*, template-based and deep-learning approaches. Our results are freely available on the EvryRNA platform at https://evryrna.ibisc.univ-evry.fr/evryrna/alphafold3/.

## Abstract 2

This article presents a comprehensive evaluation of the performance of *AlphaFold*3 in predicting 3D structures of RNA, highlighting its limitations and comparing it with ten state-of-the-art methods. Our findings, based on benchmarks from five test sets, offer valuable insights into the challenges of RNA structure prediction.

## 1. Introduction

Ribonucleic acids (RNA) are fundamental molecules that are crucial to cellular activities. While their functions are directly linked to their structures, prediction of the latter remains an open challenge to be addressed. Knowledge of the structure of RNA could be of great interest for drug design or for the comprehension of biological processes such as cancer (Zhu *et al.*, 2022 ▸). While experimental methods such as X-ray crystallography, NMR and cryo-EM can determine 3D structures of RNA, their use is costly (in terms of time and resources) and is hardly scalable to the number of RNA molecules that are found in life. Computational approaches have emerged using *ab initio*, template-based and, more recently, deep-learning methods. *Ab initio* methods (Li & Chen, 2023 ▸; Krokhotin *et al.*, 2015 ▸; Zhang, Chen *et al.*, 2021 ▸; Zhang, Li *et al.*, 2021 ▸; Boniecki *et al.*, 2016 ▸; Cragnolini *et al.*, 2015 ▸; Kerpedjiev *et al.*, 2015 ▸; Šulc *et al.*, 2014 ▸; Jonikas *et al.*, 2009 ▸; Frellsen *et al.*, 2009 ▸) tend to reproduce the physics of the system, with a force field applied to a coarse-grained representation (low resolution, in which a nucleotide is replaced by some of its atoms). Template-based approaches (Li *et al.*, 2022 ▸; Zhou *et al.*, 2022 ▸; Watkins *et al.*, 2020 ▸; Xu & Chen, 2017 ▸; Zhang, Wang *et al.*, 2022 ▸; Popenda *et al.*, 2012 ▸; Cao & Chen, 2011 ▸; Rother *et al.*, 2011 ▸; Flores *et al.*, 2010 ▸; Das & Baker, 2007 ▸) create a mapping between sequences and fragments of structure before refining the assembled structures.

With the recent success of *AlphaFold* for proteins (Senior *et al.*, 2020 ▸; Jumper *et al.*, 2021 ▸), approaches have been made to replicate its success with RNA. The direct use of protein methods to infer 3D structures of RNA is impossible, as RNA and proteins are chemically and physically different molecules. Current methods, such as *DeepFoldRNA* (Pearce *et al.*, 2022 ▸), *RhoFold* (Shen *et al.*, 2022 ▸), *DrFold* (Li *et al.*, 2023 ▸), *NuFold* (Kagaya *et al.*, 2025 ▸) and *trRosettaRNA* (Wang *et al.*, 2023 ▸), try to adapt what already exists for proteins to RNA. They consider coarse-grained representations and predict Euclidean transformations before reconstructing the full-atom structure. The use of torsio angles is also adapted to RNA, using either the standard torsion angles (Shen *et al.*, 2022 ▸; Kagaya *et al.*, 2025 ▸) or angles from their coarse-grained representations (Pearce *et al.*, 2022 ▸; Li *et al.*, 2023 ▸).

While being better than existing template-based or *ab initio* methods, deep-learning approaches do not solve the prediction of RNA structures yet, as shown in CASP-RNA (Das *et al.*, 2023 ▸) and in our recent benchmark State-of-the-RNArt (Bernard *et al.*, 2024*b* ▸). Recently, a critical review (Schneider *et al.*, 2023 ▸) explained the reasons why *AlphaFold* for RNA has not yet arrived and might not arrive for some decades. However, *AlphaFold* has released its latest version, named *AlphaFold*3 (Abramson *et al.*, 2024 ▸), that extends its predictions to different molecules, including RNA. In this work, we aim to provide a response to Schneider *et al.* (2023 ▸) in order to determine whether *AlphaFold*3 achieves success for RNA.

To extend its range of molecules, *AlphaFold*3 has made changes in its architecture in order to better adapt to the variety of available inputs. It no longer relies on torsion angles, which restricted it to specific molecules, as was the case in *AlphaFold*2 (Jumper *et al.*, 2021 ▸). It directly predicts atom coordinates with the use of a multi-cross-diffusion model. The authors mentioned good results through a benchmark on CASP-RNA (Abramson *et al.*, 2024 ▸), but *AlphaFold*3 did not outperform human-assisted methods. Furthermore, it is not clear what the current limitations are and how well it performs compared with state-of-the-art solutions.

This article aims to provide a comprehensive extension of the evaluation and benchmarking of *AlphaFold*3 for RNA. We first describe the main differences between RNA and proteins to highlight the challenges of RNA 3D structure prediction and describe the *AlphaFold*3 solution before discussing the benchmark that we performed. We then evaluate *AlphaFold*3 and comment on the results of *AlphaFold*3 and the current limitations of the model. Our benchmark also compares the performances with state-of-the-art solutions to provide a complete comparison. The results and the data are freely available and usable in the EvryRNA platform at https://evryrna.ibisc.univ-evry.fr/evryrna/alphafold3/.

## 2. RNA versus proteins

RNA and proteins are both molecules that play crucial roles in life. They share the characteristic of having a 3D structure that directly defines their function. However, it is important to acknowledge that dynamics, transient structures and unstructured proteins also play a significant role in protein function, making this relationship more complex. This section discusses the differences between RNA and proteins, highlighting the reasons why adapting existing protein models has been challenging.

RNA comprises four nucleotides (A, C, G and U), whereas proteins comprise 20 amino acids. This difference has a large consequence for the adaptation of protein algorithms to RNA. The vocabulary available for RNA is limited to four unique elements, making the protein vocabulary not directly adaptable. The sequence length of RNA molecules also has a high variability (from a dozen to thousands of nucleotides) compared with proteins (from a dozen to hundreds of amino acids).

A major difference between RNA and proteins lies in the folding stabilization. RNA structure is maintained by base pairing and base stacking, while protein structure is supported by hydrogen interactions in the skeleton. The protein backbone is also modelled by torsion angles (Φ and Ψ) for each amino acid because the peptide bond is planar. This is not the case for RNA, where each nucleotide can be described by seven torsion angles (α, β, γ, δ, ɛ, ξ and χ) and the sugar-pucker pseudorotation phase *P*. An approximation usually involves pseudo-torsions η and θ (Wadley *et al.*, 2007 ▸). However, the complexity of the RNA backbone arises not only from the number of torsional degrees of freedom but also from their intricate correlations. Specifically, the structural divergence at the phosphodiester linkage is influenced by the sugar pucker and glycosidic bond orientation of both nucleotides connected to the phosphate group. This interdependence often necessitates the description of RNA structure using dinucleotide-like fragments to accurately capture the backbone geometry (Černý *et al.*, 2020 ▸). Protein models therefore have a conformational mechanism that is fundamentally different from the RNA folding process, where such adaptations and structural dependencies must be carefully accounted for.

**Figure 1:**
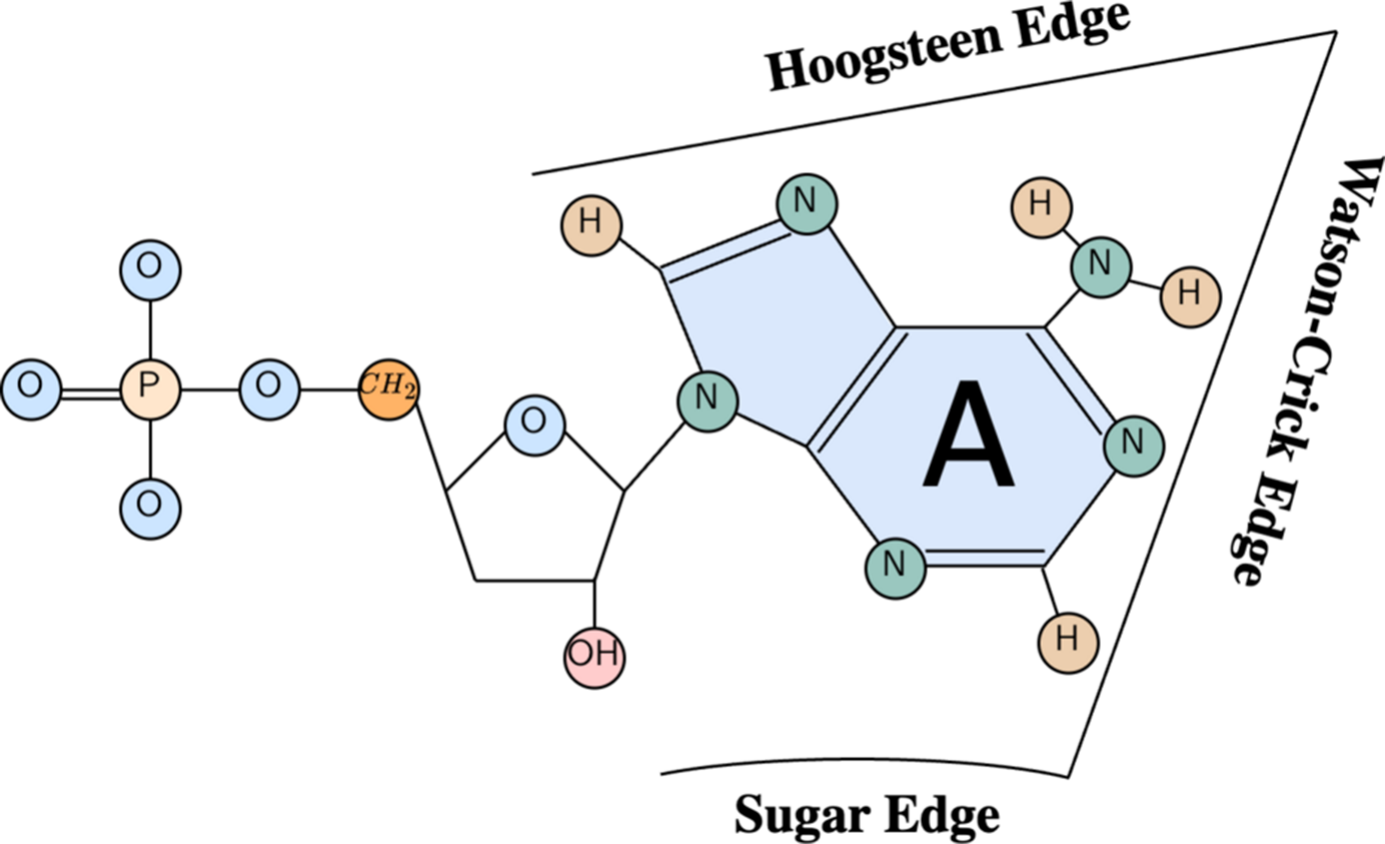
Description of the three different edges of the adenine RNA nucleotide: Watson–Crick edge, Hoogsteen edge and sugar edge. The three other nucleotides share similar edges.

The nature of pairwise interactions in 3D RNA molecules differs from those in proteins. RNA interactions can be made through three different edges of the RNA base: the WC edge, Hoogsteen edge and sugar edge (Westhof & Fritsch, 2000 ▸), as shown in Fig. 1 ▸. In addition, the orientation of the glycosidic bonds gives another property to an interaction: *cis* or *trans*. The combination of edge and orientation gives 12 possibilities for interaction between bases. The standard Watson–Crick (WC) base pair corresponds to a *cis* WC/WC pairing. Given the orientations (*cis* or *trans*), the edges and the base pairing, there are more than 200 possible base pairs. Only the standard WC pairs (*cis* WC/WC) AU and CG (and also the GU wobble pair) are used in the 2D structure representation. RNA bases also have common patterns of interactions, where a base stacks on another one. The base stacking (Gendron *et al.*, 2001 ▸; Gabb *et al.*, 1996 ▸) refers to the four base-stacking types in relative orientations (upwards, downwards, outwards and inwards; Parisien *et al.*, 2009 ▸). The extended secondary and tertiary interactions (long-range base pairs) play a crucial role in the overall topology of the RNA folding process. They help to stabilize the structure and cannot be ignored when working on 3D structures of RNA.

The stability of RNA and protein structures is different. More than five decades ago, the Nobel Prize-winning work of Christian B. Anfinsen established that, under physiological conditions, the protein chain spontaneously folds into its native structure, which is the conformation corresponding to a minimum of the Gibbs free energy that is both kinetically accessible and thermodynamically stable (Anfinsen, 1973 ▸). This native structure of the protein is also characterized by its uniqueness; although it may be altered by dynamic behaviours such as domain motions, the global fold of the protein remains the same. In contrast, RNA molecules often have a more rugged Gibbs free-energy landscape, thus populating multiple conformational states (Jang *et al.*, 2023 ▸). Switching between these conformations supports some functions of RNA, such as riboswitches or ribozymes, and may be driven by environmental changes, such as ions (notably Mg^2+^), pH, temperature or ligand binding (Yamagami *et al.*, 2021 ▸; Chheda *et al.*, 2024 ▸).

There is a huge disparity between protein and RNA data. Even if there is a higher proportion of RNA than proteins in life, this is not reflected in the available data: only a small number of 3D structures of RNA are known. Up to June 2024, 7759 RNA structures had been deposited in the Protein Data Bank (PDB; Berman *et al.*, 2000 ▸), compared with 216 212 protein structures. The quality and diversity of the data are also different: a huge proportion of RNA structures come from the same families. This implies several redundant structures that could prevent a model from being generalized to other families. In addition, a huge amount of RNA families do not yet have solved structures in the PDB, usually those of long RNA. This means there is no balanced and representative proportion of RNA families among the known structures.

Finally, no standard data set has been used for RNA throughout the community. Each research group uses a data set with different associated preprocessing. This prevents the use of deep-learning methods, as a lot of work is needed to obtain a clean data set. While the community has agreed to use RNA-Puzzles (Cruz *et al.*, 2012 ▸; Miao *et al.*, 2015 ▸, 2017 ▸, 2020 ▸) or the new CASP-RNA (Das *et al.*, 2023 ▸) to test the generalization of proposed models, no clear training set is available. The first solution was RNANet (Becquey *et al.*, 2021 ▸), which was developed in our laboratory to solve this issue. It is a database that uses MySQL and gathers diverse RNA information to train deep-learning methods. A new approach, RNA3DB (Szikszai *et al.*, 2024 ▸), creates independent data sets for deep-learning approaches, where clustering is performed based on sequence and structural disparity.

## 3. *AlphaFold*3

Building on the recent success of *AlphaFold*2 (Jumper *et al.*, 2021 ▸) in protein structure prediction, *AlphaFold*3 (Abramson *et al.*, 2024 ▸) has expanded its predictions to the structures of all molecules available in the PDB (Berman *et al.*, 2000 ▸). The authors highlight several differences from the previous architecture that contribute to successful predictions of a wide range of molecules. One key difference is the introduction of a diffusion model that reconstructs coordinates from the residue level to the atomic level. *AlphaFold*3 also directly outputs the coordinate atom positions, compared with the prediction of rotation/translation vectors (and torsion angles) in the previous version. It also weights the multiple sequence alignment (MSA) less in the overall model. In the case of RNA, *AlphaFold*3 has been evaluated on the CASP-RNA data set (Das *et al.*, 2023 ▸), demonstrating improved predictions compared with *RosettaFold*2*NA* (Baek *et al.*, 2024 ▸) and *AIchemy_RNA* (the best AI-based submission in the competition; Shen *et al.*, 2022 ▸). Despite these advancements, the performance of *AlphaFold*3 lags behind that of *AIchemy_RNA*2 (the top human-expert-aided submission; Chen *et al.*, 2023 ▸). Further details of the architecture, the training procedure and the differences between *AlphaFold*2 and *AlphaFold*3 are provided in the supporting information.

## 4. Benchmark

To assess the performance of *AlphaFold*3, we have evaluated it and compared it with other state-of-the-art methods on five data sets. This section describes the data sets and the methods as well as the metrics used to evaluate *AlphaFold*3.

### 4.1. Data sets

To evaluate the prediction of RNA structures, we considered the following five test sets, with the first three being used in our previous work (Bernard *et al.*, 2024*b* ▸).

(i) *RNA-Puzzles*. The first data set is composed of the single-stranded structures from RNA-Puzzles (Cruz *et al.*, 2012 ▸; Miao *et al.*, 2015 ▸, 2017 ▸, 2020 ▸), a community initiative to benchmark RNA structures. We considered only single-stranded solutions in order to have a fair comparison between the benchmarked models. It is composed of 22 RNAs with lengths between 27 and 188 nt (with a mean of 83 nt).
(ii) *CASP-RNA*. The second test set is composed of the CASP-RNA (Das *et al.*, 2023 ▸) structures from a collaboration between the CASP team and RNA-Puzzles. It is composed of 12 RNAs with a wide range of sequences from 30 to 720 nt (with a mean of 209 nt).
(iii) *RNASolo*. The third test set is a custom test set composed of independent structures from RNAsolo (Adamczyk *et al.*, 2022 ▸). We downloaded representative RNA molecules from RNAsolo (Adamczyk *et al.*, 2022 ▸) with resolutions below 4 Å and removed structures with a sequence identity of higher than 80%. We then considered only the structures with a unique Rfam family ID (Kalvari *et al.*, 2020), leading to 25 nonredundant RNA molecules with a sequence of between 45 and 298 nt (and a mean of 100 nt). It cannot be ensured that the structures from this data set were not used in the training sets for the different models. We keep this data set for comparison, as we already have the results for the benchmarked methods.
(iv) *RNA3DB_0*. This data set is composed of a non­redundant set of structurally and sequentially independent structures from RNA3DB (Szikszai *et al.*, 2024 ▸). It comprises the component #0, which is composed of orphan structures that are advised to be used as a test set. These structures do not belong to Rfam families (Kalvari *et al.*, 2021 ▸) and include synthetic RNAs and small messenger RNAs crystallized as part of larger complexes. After removing structures with sequences below ten nucleotides and sequence identity below 80% (using *CD-HIT*; Fu *et al.*, 2012 ▸), we ended up with a data set of 224 structures from 10 to 339 nt (with a mean of 55 nt). Nonetheless, these structures come from complexes, meaning that they do not behave well in isolation, and thus their experimentally observed conformations depend on other chains. To account for this, in evaluating the models we considered 113 structures with their full context and predicted the structures with *AlphaFold*3 (the other structures have too large a context and we failed to predict them using *AlphaFold*3). We name this subset RNA3DB_0 (Context).
(v) *RNA3DB_Long*. The last data set comprises long RNA structures from RNA3DB (Szikszai *et al.*, 2024 ▸). We considered structures with a release date after January 2023 to avoid any structure leakage for fair comparison. We considered structures with sequences between 800 nt (800 nt being the limit from previous test sets) and 5000 nt, as we wanted to study the performance of long RNAs. This led to 58 structures with a sequence of between 828 and 3619 nt (with a mean of 2005 nt). They comprise 57 ribosomal RNAs and one structure of a group II intron.

We have also ensured that all of the data sets (except RNA-Puzzles and CASP-RNA) have a sequence identity below 80% in order to have nonredundant structures for robust evaluation.

To comprehend and detail the predictions by *AlphaFold*3, we studied the three main interactions in the folding of 3D structures of RNA in detail: Watson–Crick (WC), non-Watson–Crick (nWC) and stacking (STACK). The proportion of these interactions is presented in Table 1 ▸. All data sets have the same proportion of stacking (around 75%), except for the RNA3DB_0 data set (around 56%). As RNA3DB_0 contains orphan structures, this implies structures with less common folding, as reflected by the lower proportion of stacking interactions. For all of the data sets there is a higher proportion of stacking interactions, followed by Watson–Crick and non-Watson–Crick interactions. The number of non-Watson–Crick interactions ranges from 5% to 10%, meaning that these interactions would be challenging for predictive models as they are rare in the original structures. When comparing RNA3DB_0 with or without context, we observe a greater proportion of stacking and Watson–Crick interactions in the presence of context. However, the number of non-Watson–Crick interactions remains unchanged.

### 4.2. State-of-the-art methods

Existing solutions for the prediction of 3D structures of RNA are based on three main types of methods: *ab initio*, template-based and deep-learning methods. As discussed previously in our work (Bernard *et al.*, 2024*b* ▸), *ab initio* methods (Boniecki *et al.*, 2016 ▸; Zhang, Li *et al.*, 2021 ▸; Li & Chen, 2023 ▸) usually integrate the physics of the system by simplifying the representation of nucleotides (coarse-grained). Instead of using all of the atoms for one nucleotide, they create a low-resolution representation that simplifies the computation time while losing information. They use approaches such as molecular dynamics (Qiang *et al.*, 2022 ▸) or Monte Carlo (Liu & Ou-Yang, 2005 ▸) to perform sampling in conformational space and use a force field to simulate real environmental conditions. On the other hand, template-based methods (Parisien & Major, 2008 ▸; Cao & Chen, 2011 ▸; Popenda *et al.*, 2012 ▸; Zhang, Wang *et al.*, 2022 ▸; Li *et al.*, 2022 ▸) create a mapping between sequences and known motifs with, for instance, secondary-structure trees (SSEs) before reconstructing the full structure from its sub­fragments. Finally, recent methods tend to incorporate deep-learning methods (Wang *et al.*, 2023 ▸; Kagaya *et al.*, 2025 ▸; Li *et al.*, 2023 ▸; Pearce *et al.*, 2022 ▸; Shen *et al.*, 2022 ▸) by using attention-based architectures with self-distillation and recycling, as performed in *AlphaFold*2 (Jumper *et al.*, 2021 ▸).

To compare the performance of *AlphaFold*3 (Abramson *et al.*, 2024 ▸), we benchmarked ten approaches, those used in our previous work (Bernard *et al.*, 2024*b* ▸). For the *ab initio* methods, we benchmarked *SimRNA* (Boniecki *et al.*, 2016 ▸), *IsRNA*1 (Zhang, Li *et al.*, 2021 ▸) and *RNAJP* (Li & Chen, 2023 ▸). Only *RNAJP* was used locally. For the template-based approaches, we benchmarked *MC-Sym* (Parisien & Major, 2008 ▸), *Vfold*3*D* (Cao & Chen, 2011 ▸), *RNAComposer* (Popenda *et al.*, 2012 ▸), 3*dRNA* (Zhang, Wang *et al.*, 2022 ▸) and *Vfold-Pipeline* (Li *et al.*, 2022 ▸). For the deep-learning methods, we benchmarked *trRosettaRNA* (Wang *et al.*, 2023 ▸) and *RhoFold* (Shen *et al.*, 2022 ▸). Further details of each method have been provided in our previous article (Bernard *et al.*, 2024*b* ▸). For RNA-Puzzles and CASP-RNA, we included the predictions from the official results of the competitions in the benchmark. We refer to these as ‘challenge best’ and they correspond to different methods for each RNA. We normalized each prediction using *RNA-tools* (Magnus *et al.*, 2020 ▸) to give a standard format for all structures. It gives standardized names for chains, residues and atoms and removes ions and water.

We used the web servers with default parameters to compare available models fairly, so that users could reproduce our experiments. As we made most of the predictions using web servers, the predictions for RNA3DB_0 were hardly applicable to all of the methods. Therefore, we benchmarked the RNA3DB_0 data set with one method per approach (the quickest method per approach): *RhoFold* (Shen *et al.*, 2022 ▸) for deep learning, *RNAComposer* (Popenda *et al.*, 2012 ▸) for template-based and *RNAJP* (Li & Chen, 2023 ▸) for *ab initio*. For the RNA3DB_Long data set, only *AlphaFold*3 could predict structures with sequences up to 3000 nt. For this data set, we only considered the predictions from *AlphaFold*3.

### 4.3. Evaluation metrics

To compare the predictions, we used the *RNAdvisor* tool developed by our team (Bernard *et al.*, 2024*a* ▸), which enables the computation of a wide range of existing metrics on one command line. For the evaluation of 3D structures of RNA, a general assessment of the folding of the structure can be performed with either the root-mean-square deviation (RMSD) or its extension adding RNA features ɛRMSD (Bottaro *et al.*, 2014 ▸). Protein-inspired metrics can also be adapted to assess structure quality, such as the TM-score (Zhang & Skolnick, 2004 ▸; Gong *et al.*, 2019 ▸) or the GDT-TS (which counts the number of superimposed atoms; Zemla *et al.*, 1999 ▸). There are also the CAD-score (which measures the structural similarity in a contact-area difference-based function; Olechnovič *et al.*, 2013 ▸) and the lDDT (which assesses the interatomic distance differences between a reference structure and a predicted structure; Mariani *et al.*, 2013 ▸). Finally, RNA-specific metrics have been developed, such as the *P*-value (which assesses the non-randomness of a given prediction; Hajdin *et al.*, 2010 ▸). The INF-ALL (Parisien *et al.*, 2009 ▸) and DI (Parisien *et al.*, 2009 ▸) have been developed to consider RNA-specific interactions. The INF score incorporates canonical and noncanonical pairing with Watson–Crick (INF-WC), non-Watson–Crick (INF-NWC) and stacking (INF-STACK) interactions. The consideration of torsion angles has been developed with the mean of circular quantities (MCQ; Zok *et al.*, 2014 ▸) and LCS-TA (longest continuous segments in torsion angle space; Wiedemann *et al.*, 2017 ▸). As discussed in Bernard *et al.* (2024*a* ▸), all of these metrics are complementary and can infer different aspects of RNA 3D structure behaviour. For the rest of the article, we will discuss the RMSD, INF-ALL, lDDT, TM-score and MCQ; the results for the other metrics are given in the supporting information. Indeed, the RMSD is the most used metric in the literature, and the INF-ALL incorporates key RNA interactions. The lDDT and TM-score allow the evaluation of global conformations (widely used in *AlphaFold*3), and MCQ gives the torsional deviation. We only mention all of the metrics when comparing the different models to ensure a complete evaluation.

## 5. Results

This section presents the results of *AlphaFold*3 predictions on the discussed test sets. We start by comparing the results of *AlphaFold*3 with existing solutions and then discuss in detail the link between the performance and the sequence length. Next, we discuss the results of *AlphaFold*3 on ribosomal structures (RNA3DB_Long data set) and orphan structures (RNA3DB_0 data set). We then discuss the results of specific RNA key interactions in detail before shedding light on the computation time.

### 5.1. *AlphaFold*3 compared with the state of the art

**Figure 2:**
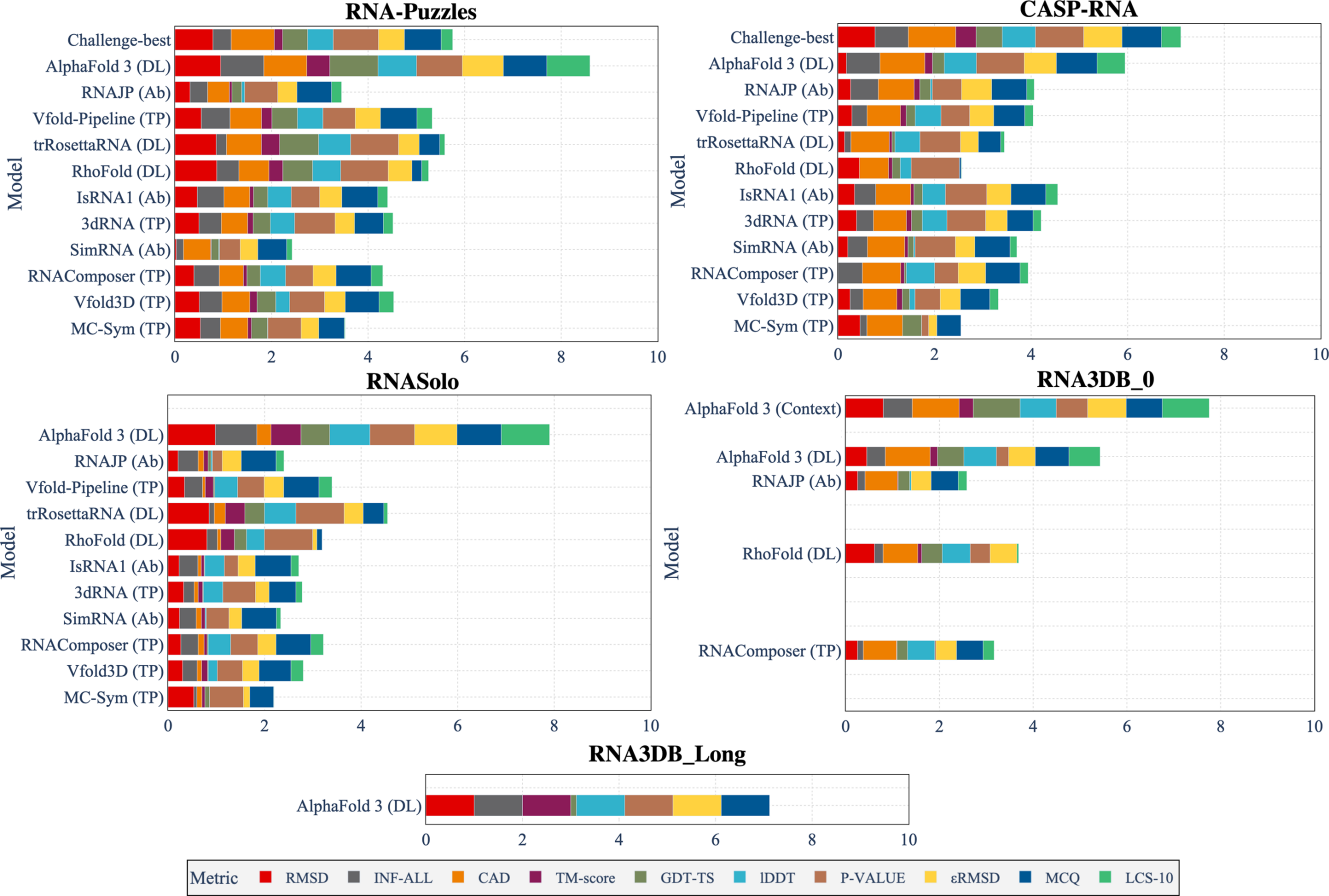
Cumulative normalized metrics (the higher the better) for each of the benchmarked methods for our five test sets. Each metric is normalized by the maximum value over the five test sets, and the decreased metrics are inverted to have better values close to 1. Challenge-best means the best solutions from the RNA-Puzzles and CASP-RNA competitions (and corresponds to different solutions for each challenge). The types of methods are also mentioned with the abbreviations DL for deep learning, TP for template-based and Ab for *ab initio*. Methods are sorted by release time (except for challenge-best). *AlphaFold*3 (Context) represents the predictions of *AlphaFold*3 for 113 structures of the RNA3DB_0 data set with the context of the structures added as input.

We compare the predictions of the ten existing methods presented above and *AlphaFold*3 on our different test sets. Fig. 2 ▸ presents the different normalized metrics computed for the prediction of the different models over the five test sets. We included all metrics to show the cumulative performance. The RNA3DB_Long data set only has predictions from *AlphaFold*3, which is the only method that is capable of processing long sequences. All of the metrics are normalized by the maximum values and converted to be better when near to 1 and worse when near to 0. Real values for each metric for the five test sets are reported in Supplementary Tables S1, S2, S3, S4 and S5.

The best models from the CASP-RNA competition, which are human-guided, outperform *AlphaFold*3 (*p*-value = 0.007; Wilcoxon signed-rank test) for every metric (except for LCS-TA, with a threshold of 10°, and MCQ) for the CASP-RNA data set. On the other hand, *AlphaFold*3 shows a cumulative sum of metrics greater than the other methods for the other test sets (*p*-value < 10^−5^ for RNA-Puzzles, *p*-value < 10^−4^ for RNASolo). For RNA-Puzzles, the challenge-best solutions are from older solutions with less advanced architectures compared with the more recent CASP-RNA solutions. For the RNA3DB_0 data set, the performance of *AlphaFold*3 is slightly better compared with *RhoFold*, which gives a better RMSD but a worse MCQ and LCS-TA. *AlphaFold*3 always has a high MCQ value, indicating that it returns structures which are more physically plausible than *ab initio* methods (which use physics properties in their predictions). Nonetheless, it does not always have the best RMSD (outperformed in CASP-RNA and RNA3DB_0), suggesting that *AlphaFold*3 does not always have the best alignment (in terms of all atoms) compared with the reference structure.

**Figure 3:**
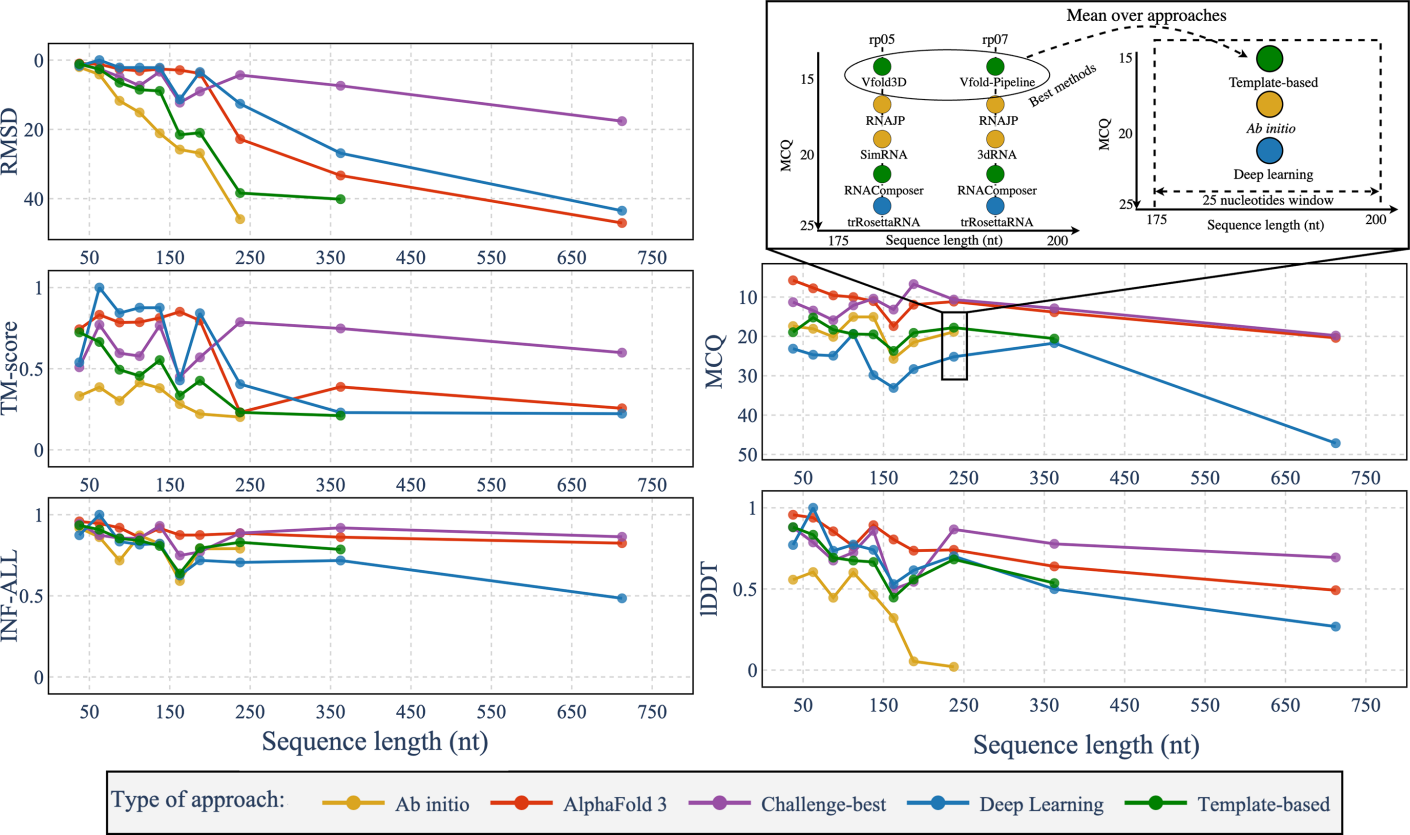
Averaged metrics depending on the sequence length for the different approaches (*AlphaFold*3, *ab initio*, deep-learning, template-based and challenge-best). Each point represents the metric averaged over the best models of each approach for a window of 25 nt from 25 to 750 nt. *Ab initio* methods group *RNAJP*, *IsRNA*1 and *SimRNA*, while template-based methods group *Vfold-Pipeline*, 3*dRNA*, *RNAComposer*, *Vfold*3*D* and *MC-Sym*. Deep-learning methods group *trRosettaRNA* and *RhoFold*. Metrics are computed for the RNA-Puzzles, CASP-RNA and RNASolo data sets. Challenge-best corresponds to the best results from either the RNA-Puzzles or CASP-RNA competitions but does not appear for the RNASolo data set. The metrics are RMSD, MCQ, TM-score, lDDT and INF-ALL. RMSD and MCQ are reversed to have the best values near the top and the worst values at the bottom.

To compare the global performance of each type of approach, in Fig. 3 ▸ we report the averaged metrics over the different types of approach depending on the sequence length. We grouped the results for structures in a sequence-length window of 25 nt (each point represents the mean computed on the best results per approach with sequence length from this 25-nucleotide window). Results of the other metrics are shown in Supplementary Fig. S2. None of the benchmarked *ab initio* methods successfully predicted structures for sequences exceeding 200 nt, particularly when using web servers. The best results from the CASP-RNA and RNA-Puzzles challenges outperform *AlphaFold*3 across most metrics, except for sequences between 150 and 250 nt, where *AlphaFold*3 showed comparable results. The values of RMSD, TM-score, MCQ and lDDT tend to worsen with sequence length, reflecting a general trend of loss of accuracy with longer RNA structures. For INF, there is no clear degradation tendency, meaning that the reproduction of the interactions does not have a strong link to the sequence length. *Ab initio* and template-based methods have competitive MCQ values, while *ab initio* methods tend to have a global alignment that is worse than the other methods (due to the high simulation time, which is a bottleneck for web-server usage). Deep-learning approaches, in particular, produced worse MCQ scores than traditional methods. *AlphaFold*3 demonstrated an especially strong MCQ performance, with comparative results for the best solutions of challenges for sequences greater than 250 nt.

These results suggest that *AlphaFold*3 achieves a competitive performance, particularly in capturing more realistic torsion angles through better MCQ scores (which is not the case for other existing deep-learning methods), although it remains outperformed by global assessment for structures of more than 200 nt.

### 5.2. The performance of *AlphaFold*3 relative to sequence length

**Figure 4:**
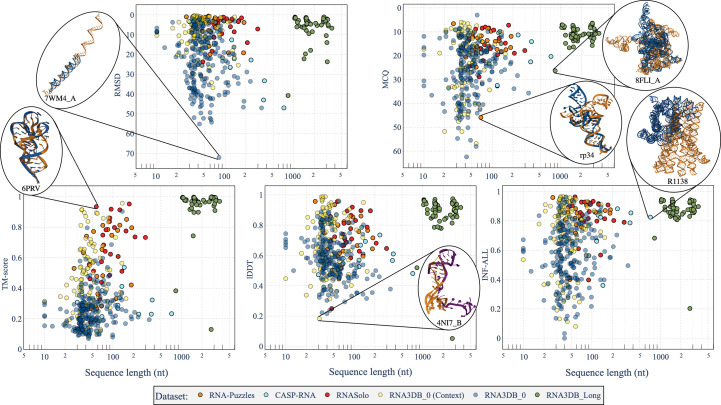
Dependence of metrics on the sequence length in predictions of *AlphaFold*3 (Abramson *et al.*, 2024 ▸) on the five test sets. For some of the predictions, we show the predicted structure (in blue or purple if predicted using context) aligned with the native structure (in orange) using *US-align* (Zhang, Shine *et al.*, 2022 ▸). The metrics are RMSD, MCQ (Zok *et al.*, 2014 ▸), TM-score (Zhang & Skolnick, 2004 ▸; Zhang, Shine *et al.*, 2022 ▸), lDDT (Mariani *et al.*, 2013 ▸) and INF-ALL (Parisien *et al.*, 2009 ▸). RMSD and MCQ are reversed to have the best values near the top and the worst values at the bottom.

As seen previously, the prediction of 3D structures of RNA usually becomes harder when the sequence length increases. Indeed, the *ab initio* methods fail to predict long interactions as the computation time increases greatly with sequence length. The template-based approaches, as well as the deep-learning methods, are limited by the small number of long RNA structures, as shown in Bernard *et al.* (2024*b* ▸). To observe the relation between sequence length and *AlphaFold*3 performance in more detail, in Fig. 4 ▸ we report the RMSD, MCQ, TM-score, lDDT and INF-ALL metrics depending on sequence length (for the five test sets). The links between the other metrics and the sequence length are available in Supplementary Fig. S3.

Fig. 4 ▸ indicates that, except for the RNA3DB_0 data set, the RMSD becomes worse for sequences between 0 and 1000 nt. For the RNA3DB_Long data set with sequences longer than 1000 nt the predictions have good results for every metric. We also observe a tendency for degradation in the lDDT, TM-score and INF-ALL (smaller decrease) when the structures have sequences of longer than 100 nt (and below 1000 nt). For every metric, the predictions for the RNA3DB_0 (with or without context) data set seem to have no clear dependence on the sequence length. For the other test sets with structures with sequences between 200 and 1000 nt, there is a common tendency to worsen in terms of performance for the *AlphaFold*3 predictions.

### 5.3. *AlphaFold*3 results on long RNA

Current methods for the prediction of RNA 3D structures are limited for long RNA and hardly predict structures with sequences longer than 200 nt. *AlphaFold*3 is, to the best of our knowledge, the only method that can predict long RNA structures (with sequences longer than 1000 nt). Its predictions on RNA3DB_Long show a good performance, as shown in Fig. 2 ▸. The only metrics for which the results are not good are GDT-TS, CAD-score and LCS-TA (threshold of 10°), which might be due to an error in computation. For LCS-TA, the low score could be explained by the difficulty of keeping a low MCQ for a high proportion of the structure, as the sequences are long for this data set.

The good results for long RNA can be explained by the types of structures used in RNA3DB_Long. Indeed, all of the structures (except for one) are ribosomal RNAs and thus they have a high redundancy. This might be reflected in the PDB, which has been memorized by *AlphaFold*3 during its training. As *AlphaFold*3 uses the MSA as inputs, it could find similarities with trained structures and thus return excellent predictions if the families are well known. Most of the long RNAs in the PDB share common structures in the ribosomal family. Therefore, these results show a good generalization of previously observed families from *AlphaFold*3.

**Figure 5:**
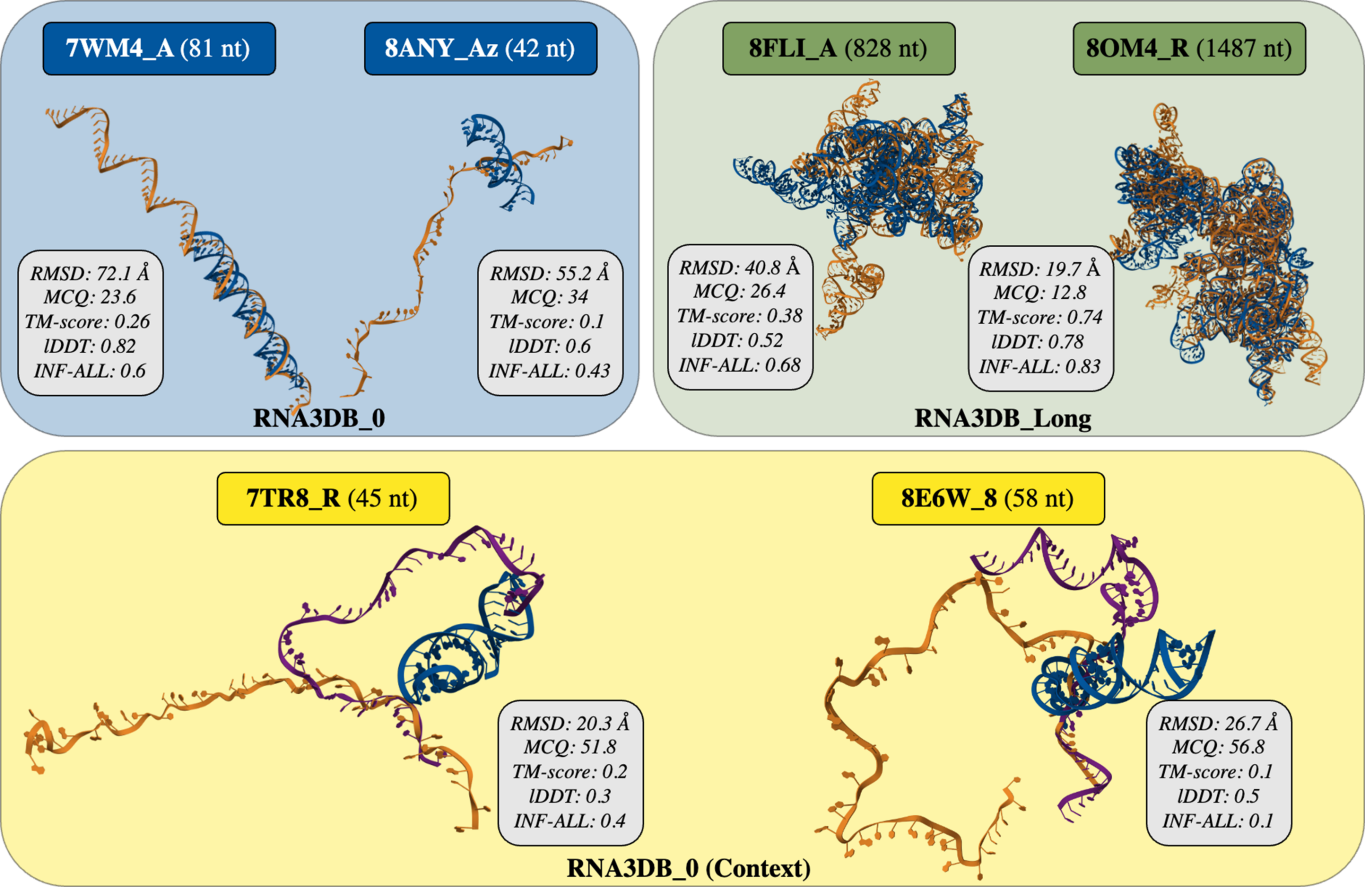
The worst two predicted structures (based on a cumulative sum of metrics) from *AlphaFold*3 (Abramson *et al.*, 2024 ▸) for the RNA3DB_0 (left), RNA3DB_Long (right) and RNA3DB_0 (Context) (bottom) data sets. The RMSD, MCQ (Zok *et al.*, 2014 ▸), TM-score (Zhang & Skolnick, 2004 ▸; Zhang, Shine *et al.*, 2022 ▸), lDDT (Mariani *et al.*, 2013 ▸) and INF-ALL (Parisien *et al.*, 2009 ▸) are provided for each structure. The predictions from *AlphaFold*3 (in blue) are aligned with the native structures (in orange) using *US-align* (Zhang, Shine *et al.*, 2022 ▸). The predictions of *AlphaFold*3 with context [only for RNA3DB_0 (Context)] are provided in purple.

We report the two worst predictions of *AlphaFold*3 on the RNA3DB_Long data set in Fig. 5 ▸. The two worst predictions for the other test sets are provided in Supplementary Fig. S4. The RMSD for the two structures is relatively high (greater than 19 Å). The second worst structure has a high TM-score (0.74), meaning that even for a long structure (1487 nt) the global alignment of atoms is well predicted. INF-ALL is also high for these structures (higher than 0.68), meaning that it returns a high proportion of key RNA interactions. In detail, it is most likely to be no coincidence that the worst prediction (TM-score = 0.38) corresponds to the only nonribosomal RNA in the RNA3DB_Long data set, while the overwhelming majority of available native structures for long RNA sequences belong to ribosomes. In addition, the lack of structural context did not help *AlphaFold*3 either, as this group II intron RNA can be found in complex with its large maturase/reverse transcriptase (PDB entry 8fli; Haack *et al.*, 2024 ▸). The medium-to-high quality of the second-worst prediction (TM-score = 0.74) can be explained by the fact that it occurred for the 15S mitochondrial ribosomal RNA (PDB entry 8om4; Ast *et al.*, 2024 ▸). This RNA is analogous, yet evolutionarily distant, from its bacterial and eukaryotic counterparts (the 16S and 18S RNAs, respectively) and its 3D structure has rarely been studied; it has been reported in only three articles (Desai *et al.*, 2017 ▸; Harper *et al.*, 2023 ▸; Ast *et al.*, 2024 ▸).

### 5.4. *AlphaFold*3 results on orphan structures

The RNA3DB_0 data set is mainly composed of structures without any hit in the Rfam family, and thus contains orphan structures. The results of *AlphaFold*3 for this data set, as presented in Figs. 2 ▸ and 4 ▸, show an overall lower performance compared with the other data sets when there is no use of context. *AlphaFold*3 performs slightly better than *RhoFold* for this data set (*p*-value = 0.015). When using context, *AlphaFold*3 produces improved results compared with those without context (*p*-value < 10^−19^).

We detail the two worst predictions for RNA3DB_0 and RNA3DB_0 (Context) from *AlphaFold*3 in Fig. 5 ▸. We observe poor results in terms of metrics (high RMSD and MCQ values and low TM-score and INF-ALL) for the two structures without context. With context, *AlphaFold*3 seems to understand that the predictions are not only helices but still fails in these two worst examples to predict the complex non-common folding of these RNAs. These structures also have a small number of nucleotides (81, 42, 45 and 58 nt), meaning that *AlphaFold*3 might not fail because of long-range interactions. Instead, these structures do not have a known family and rely on a complex environment of other molecules. With context, *AlphaFold*3 has a better chance of predicting the structural folding well, but the generalization is not always robust for structures without known families, even with small structures (as shown by the mean value of TM-score, which is less than 0.5, in Supplementary Table S4).

**Figure 6:**
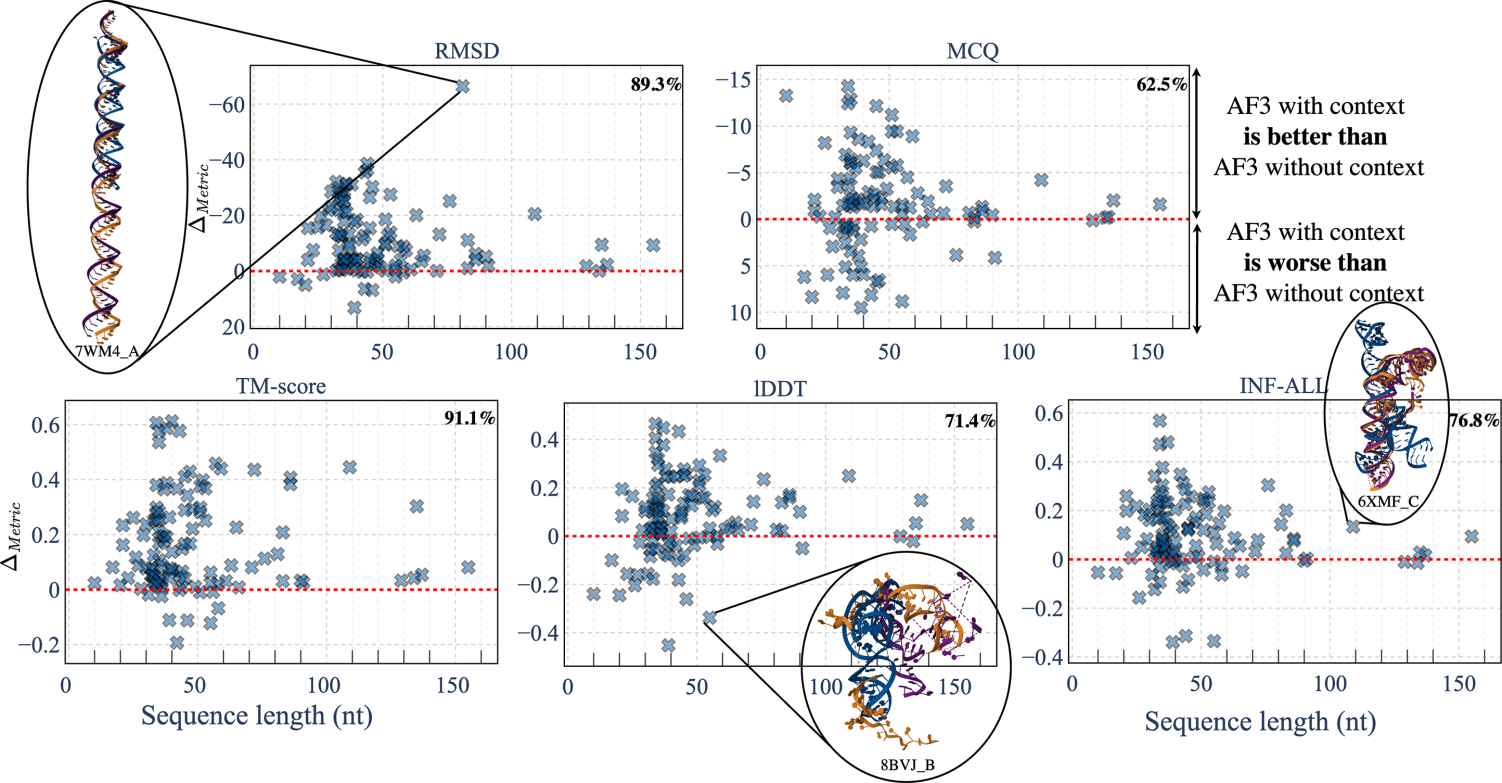
Difference per metric between results from *AlphaFold*3 with context and without context for the common structures of the RNA3DB_0 data set depending on the sequence length. Regions above the red line correspond to structures where the results from *AlphaFold*3 with context are better than those without context. We reversed the RMSD and MCQ metrics so that higher regions always depict the same behaviour. The percentage of cases where *AlphaFold*3 with context outperforms predictions without context is reported in the top-right corner of each plot. Structures are reported with the native structure in orange, predictions with *AlphaFold*3 without context in blue and with context in purple.

To further study the impact of context for the prediction of RNA structures, in Fig. 6 ▸ we report the differences per metric between predictions of *AlphaFold*3 with and without context depending on the sequence length. Details of each metric value for each RNA are provided in Supplementary Fig. S7. For all metrics, there is an improvement on using context: 91.1% of structures with context have a better TM-score than those without context. For the MCQ metric, 62.5% of structures with context outperform those without context, which is less dominant than for the other metrics. For example, in the case of PDB entry 7wm4 (Sakaniwa *et al.*, 2023 ▸), the context effectively facilitates the identification of the correct scale for one half of the double helix. Similarly, for PDB entry 8bvj (Dendooven *et al.*, 2023 ▸), which features a discontinuity, the context enables *AlphaFold*3 to accurately detect the discontinuities. However, this does not result in better alignment in terms of the lDDT metric. Incorporating contextual information significantly enhances the global alignment performance, as reflected by improvements in the RMSD, TM-score and lDDT metrics. This is followed by moderately smaller, but still notable, improvements in reproducing key RNA interactions (INF metric) and torsion angles (MCQ metric). Among the benchmarked models, the possibility of using context in the prediction is only available with *AlphaFold*3. The other models are specialized for RNA and are not designed to process different molecules.

### 5.5. *AlphaFold*3 results on key RNA interactions

**Figure 7:**
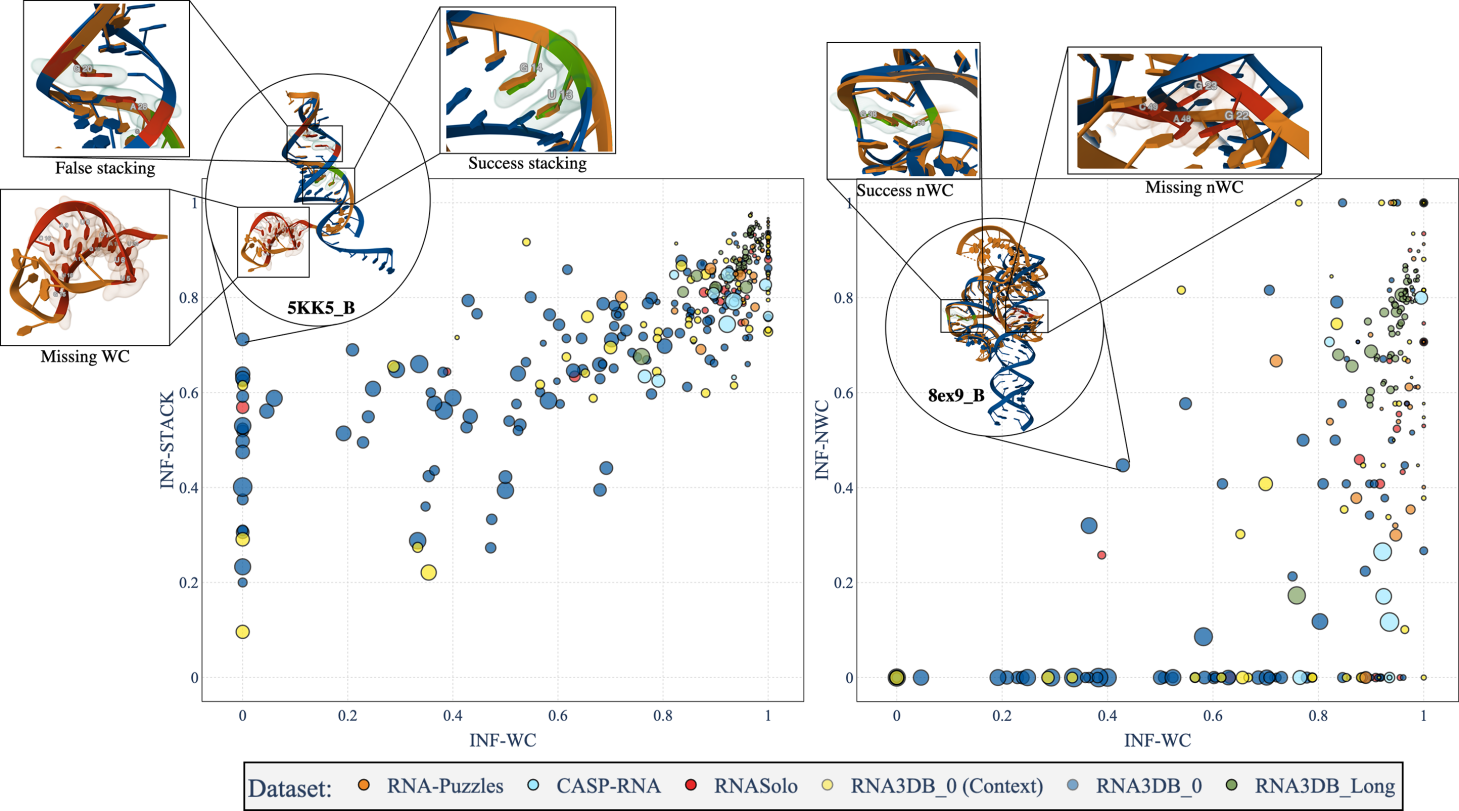
Link between INF Watson–Crick (WC) and non-Watson–Crick (nWC) and stacking (STACK) interactions in the predictions of *AlphaFold*3 for our five test sets. The area of each point is proportional to the RMSD: the lower the better. Only structures with at least one non-Watson–Crick interaction are shown in the figures. An INF (Parisien *et al.*, 2009 ▸) value of 1 means accurate reproduction of key RNA interactions, while a value near 0 means that the structure does not reproduce the interactions. Left: INF stacking (INF-STACK) depending on INF Watson–Crick (INF-WC) interactions. Right: INF non-Watson–Crick (INF-nWC) depending on INF Watson–Crick (INF-WC) interactions.

To evaluate the ability of *AlphaFold*3 to predict noncanonical interactions, we depict the scatter plots between non-Watson–Crick INF (INF-NWC) and Watson–Crick INF (INF-WC) in Fig. 7 ▸. The size of the points is proportional to the RMSD of the structures and thus to their global atom alignment. We observe a tendency to have a low RMSD (small points) whenever the INF-WC and INF-NWC are high. There are also many structures with an INF-NWC of 0, suggesting that *AlphaFold*3 does not predict any of the non-Watson–Crick interactions (mostly for the RNA3DB data set). Examples of successful and missing non-Watson–Crick interactions are shown in the figure. For the results on stacking interactions, there are predictions where *AlphaFold*3 does not predict the Watson–Crick interactions well but still predicts the stacking interactions. This can be explained by good skeleton predictions while lacking the base conformations that produce the WC interactions. Secondly, there is an increased correlation between INF-STACK and INF-WC: when *AlphaFold*3 predicts the WC interactions well, it also tends to estimate the stacking well. Indeed, the stacking interactions tend to align with the correct base pairing, but the correlation is likely to be influenced by whether the sequence can fold into the observed conformation. For instance, in Fig. 7 ▸, parts of PDB entry 8ex9 chain *B* can fold, whereas others cannot.

**Figure 8:**
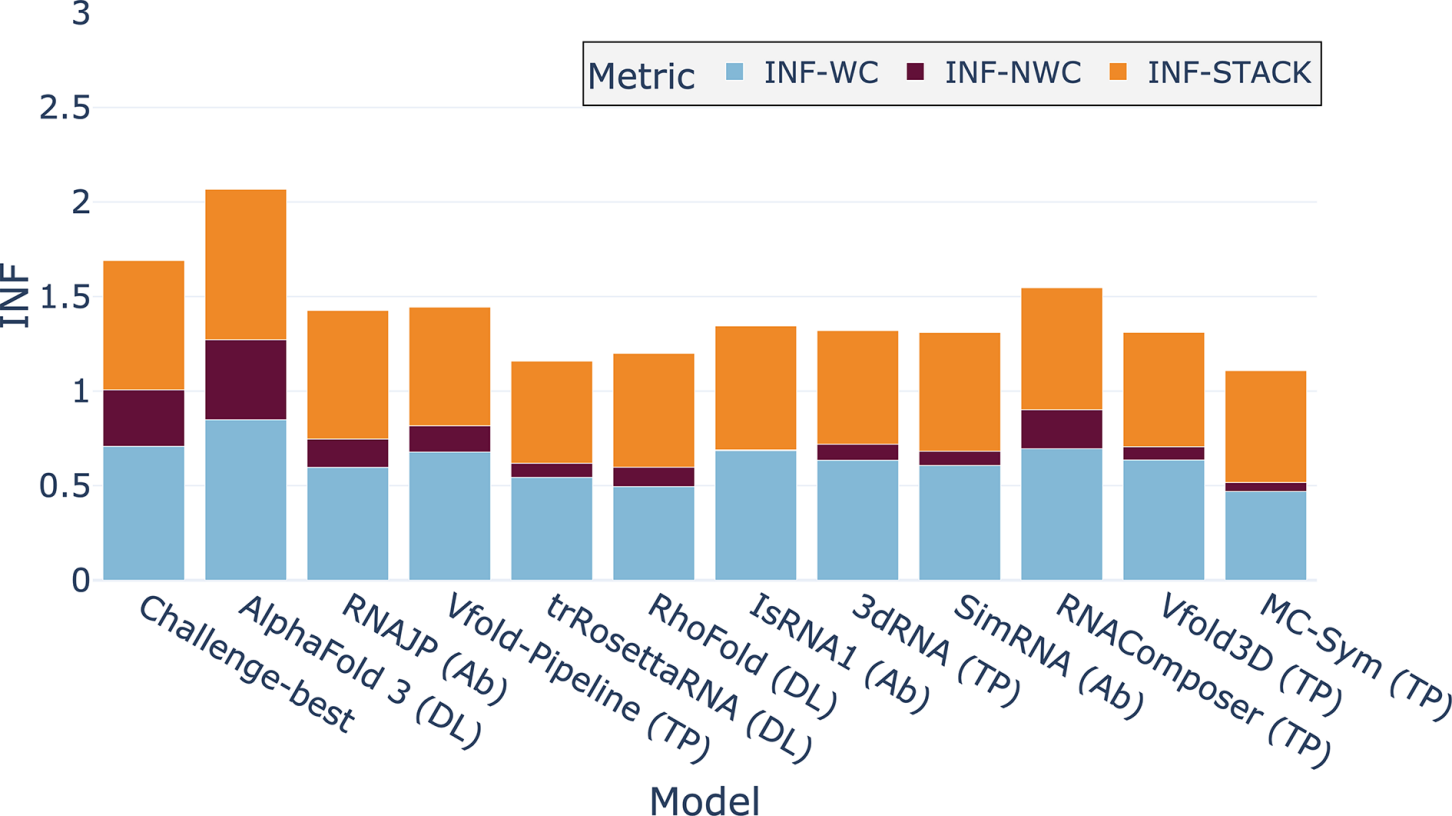
INF metrics for the different benchmarked models averaged over three test sets: RNA-Puzzles, CASP-RNA and RNASolo. INF metrics consider Watson–Crick (INF-WC), non-Watson–Crick (INF-NWC) and stacking (INF-STACK) interactions.

To compare the key RNA interactions predicted from *AlphaFold*3 with existing solutions, in Fig. 8 ▸ we present the mean INF metrics (INF-WC, INF-NWC and INF-STACK) over RNA-Puzzles, CASP-RNA and RNASolo for the ten benchmarked models. Details for each data set are provided in Supplementary Table S6. We only show the results on these data sets as we only had complete predictions for each model for these three data sets. *AlphaFold*3 has better values for each INF metric compared with the other methods. The second-best method to reproduce RNA key interactions is *RNAComposer*. While having good overall results in terms of cumulative metrics,* trRosettaRNA* shows poor results in terms of key RNA interactions. Even if *AlphaFold*3 outperforms other solutions for all of the INF metrics, the results for nWC interactions remain low (below 0.5), meaning that progress is still needed to reproduce RNA-specific interactions well.

### 5.6. Computation time

*AlphaFold*3 is a deep-learning method that has a complex architecture. Compared with existing *ab initio* methods, deep-learning methods tend to be faster for inference. We report the computation time for a small RNA molecule (27 nt) and a long RNA moelcule (434 nt) for *RNAComposer* (Popenda *et al.*, 2012 ▸), *RhoFold* (Shen *et al.*, 2022 ▸), *trRosettaRNA* (Wang *et al.*, 2023 ▸), *RNAJP* (Li & Chen, 2023 ▸) and *AlphaFold*3 (Abramson *et al.*, 2024 ▸) in Table 2 ▸. We report the computation time for the fastest methods, while the times for the rest of the methods are available in our previous work (Bernard *et al.*, 2024*b* ▸). As we could only run *RNAJP* locally and each web server has different configurations, there is a bias in the comparison. *RNAComposer*, *RhoFold* and *trRosettaRNA* all predict small RNA very quickly (in less than a minute), while *RNAJP* takes 2 h (with default parameters). For a structure with a longer sequence, it is *RNAComposer* that has the fastest computation time (around 3 min). The *ab initio* method *RNAJP* takes 15 h. *AlphaFold*3 returns a prediction in around 5 min, which shows fast inference. For RNA with very long sequences (around 3000 nt), *AlphaFold*3 take multiple hours to predict (and sometimes returns errors and needs to be run multiple times to obtain results).

## 6. Discussion

*AlphaFold*3 is a deep-learning method that has widened its scope to predict RNA structures (as well as other molecules) compared with its previous approach. Through our benchmark, we showed that *AlphaFold*3 is a competitive method that outperforms most of the existing solutions. It yields better results for RNA-Puzzles and RNASolo, but remains outperformed by the best solutions from the CASP-RNA challenge.

*AlphaFold*3 has achieved good generalization properties for ribosomal structures (RNA3DB_Long data set). This shows bias from the existing data for RNA: most of the long RNA structures available in the PDB are of ribosomal-related RNA.

*AlphaFold*3 returns results with an overall good reproduction of key RNA interactions compared with existing solutions. It is also the best method to reproduce RNA torsion angles (best results in terms of MCQ), which was lacking in the existing deep-learning methods (Bernard *et al.*, 2024*b* ▸).

There remain limitations that need to be addressed regarding the RNA folding problem. *AlphaFold*3 does not reproduce all of the non-Watson–Crick interactions, which is essential for the stability of 3D RNA structures. Furthermore, *AlphaFold*3 fails to predict structures from orphan families (RNA3DB_0 data set) without context. These structures are hard to predict as there is no hint in the available data, and reliable information is often supported by the context and the environment of the RNA. *AlphaFold*3 achieves better results when providing the context, but there remains a limitation of generalization for these orphan RNAs in our evaluation. Evaluating orphan structures remains challenging, as environmental information or context is lacking. There is also no easy way to correctly evaluate the alternative solutions proposed by *AlphaFold*3, whereas multiple conformations are possible for RNA. *AlphaFold*3, while reducing the impact of the MSA on its architecture, still uses it, restricting its scope for RNA (as there are still unknown families). The computation time for the inference is very fast but remains limited by its usage in web servers. The source code has been released but requires huge computational resources to be easily used.

## 7. Conclusion

*AlphaFold*2 has had huge success in the prediction of protein folding and has changed the field by the quality of its predictions. The new release of *AlphaFold*, named *AlphaFold*3, has extended the model to predict all molecules in the PDB, such as ions, ligands, DNA and RNA.

Through an extensive benchmark on five different test sets, we have evaluated the quality of predictions of *AlphaFold*3 for RNA molecules. We have also compared the results with ten existing methods, which are easily reproducible as their predictions are available using web servers.

Our results show that *AlphaFold*3 is of competitive quality, as it outperforms most of the existing solutions. It returns more physically plausible structures than *ab initio* methods. It outclasses existing deep-learning approaches for every data set while better reproducing key RNA interactions and torsion angles. It also returns predictions very quickly compared with *ab initio* or current template-based approaches [but does not exceed *RNAComposer* (Popenda *et al.*, 2012 ▸) for inference time].

For ribosomal long RNAs, *AlphaFold*3 returns highly accurate predictions. This could be explained by its capability to generate structures from known families which have been seen in its training data. As there are not a lot of data available, it is difficult to find complex structures without any homologs to evaluate performances fairly.

Nonetheless, *AlphaFold*3 has not yet covered RNA with the same success as it has proteins. Its new architecture allows the prediction of a wide range of molecules but remains limited and hardly predicts non-Watson–Crick interactions. It does not generalize well to orphan structures which are not related to any known RNA families. Prediction of these structures requires knowledge of the context, which it is possible to integrate with *AlphaFold*3.

The prediction of atom coordinates instead of base frames, as performed in *AlphaFold*2, allows the extension of predictions to a wide range of molecules but prevents the generalization of RNA-specific interactions. The lack of data is also a limitation that prevents the robustness of deep-learning methods in general, including *AlphaFold*3.

## 8. Related literature

The following references are cited in the supporting information for this article: Evans *et al.* (2021 ▸), Ji *et al.* (2023 ▸), RNA Consortium (2021 ▸), Sayers *et al.* (2023 ▸) and Sha *et al.* (2023 ▸).

## Supplementary Material

<it>AlphaFold</it>3 architecture, training procedure, differences from previous approaches and limitations, and Supplementary Figures and Tables. DOI: 10.1107/S2059798325000592/lie5001sup1.pdf

## Figures and Tables

**Table 1:** Proportion of key RNA interactions in the five test sets (and the subset of RNA3DB with context) The interactions are normalized by the number of residues. Interactions are either stacking (STACK), Watson–Crick (WC) or non-Watson–Crick (nWC), as extracted from MC-Annotate (Gendron *et al.*, 2001 ▸). Interactions for RNA3DB_0 are computed without context, while RNA3DB_0 (C) includes context.

Interaction type	STACK	WC	nWC
RNA-Puzzles	0.78	0.33	0.10
CASP-RNA	0.75	0.35	0.05
RNASolo	0.77	0.31	0.09
RNA3DB_0	0.56	0.14	0.04
RNA3DB_0 (C)	0.61	0.16	0.04
RNA3DB_Long	0.74	0.29	0.10

**Table 2:** Computation time for sequences of 27 nt (PDB entry 6y0y; E. Ennifar & E. Westhof, unpublished work) and 434 nt (PDB entry 7xd6; Luo *et al.*, 2023 ▸) Computation time is computed using web servers except for *RNAJP*. Methods are sorted by release time. The types of approaches are either template-based (TP), *ab initio* (Ab) or deep learning (DL).

Model	Approach	Time (27 nt)	Time (434 nt)
*RNAComposer* (Popenda *et al.*, 2012 ▸)	TP	1	3
*RhoFold* (Shen *et al.*, 2022 ▸)	DL	1	10
*trRosettaRNA *(Wang *et al.*, 2023 ▸)	DL	1	600
*RNAJP*† (Li & Chen, 2023 ▸)	Ab	120	900
*AlphaFold*3 (Abramson *et al.*, 2024 ▸)	DL	2	5

† *RNAJP* computation time is computed locally with a simulation time set to 50 × 10^6^ steps on an NVIDIA P1000.
