# Supplementary material for: Has AlphaFold3 achieved success for RNA?
Source: Acta Crystallogr D Struct Biol. 2025 Jan 27;81(Pt 2):49–62. doi: 10.1107/S2059798325000592 (PMC11804252; doi:10.1107/S2059798325000592)
Supplement: Supplementary file 1 [file d-81-00049-sup1.pdf]

STRUCTURAL
BIOLOGY

Volume 81 (2025)

Supporting information for article:

Has *AlphaFold*3 reached its success for RNA?

Clément Bernard, Guillaume Postic, Sahar Ghannay and Fariza Tahi

Has AlphaFold 3 reached its success for RNAs? Supplementary File

AlphaFold 3

In this section, we present briefly the AlphaFold 3 architecture, the training procedure, the differences from previous approaches and the limitations mentioned in the article.

AlphaFold 3 architecture

AlphaFold 3 takes as inputs a sequence of a given molecule (amino acids for proteins, nucleotides for RNAs, etc) and embeds it to different main blocks: the input embedder, the pairformer and the diffusion module. The sequence input is represented as tokens, where a token is considered a nucleotide for RNAs. An MSA module is added before the Pairformer, which reduces its overall importance in the network. A different input now embeds the information, the pair representation. The architecture also adds confidence measures like pLDDT (modified local distance difference test), PAE (predicted aligned error) and PDE (distance error matrix).

A novelty brought by the third version of AlphaFold 3 is the replacement of the structural module with a diffusion module. This module trains a denoiser to remove Gaussian noise from coarse-grained representation (one atom per nucleotide for RNA/DNA or per amino acid for protein) to full atom representation. No explicit geometry is involved, which differs from existing recent solutions (Sha *et al.*, 2023; Li *et al.*, 2023; Shen *et al.*, 2022; Wang *et al.*, 2023; Kagaya *et al.*, 2023). Instead, the diffusion module is trained to reconstruct 48 different versions of the reference structure (randomly rotating, translating, and noised). The training loss for the diffusion module is a weighted aligned MSE loss, which is then incremented by a structure-based loss based on smooth LDDT for the fine-tuning part.

The final architecture of AlphaFold 3 can predict up to 5120 tokens, for an inference time of 347 seconds on 16 NVIDIA A100 GPUs (Abramson *et al.*, 2024).

Training procedure

AlphaFold 3 was trained using a mixture of five datasets (PDB structures, transcription factors and distilled datasets). The RNA data came from the Rfam (Kalvari *et al.*, 2020), RNACentral (Consortium, 2021), and Nucleotide collection (Sayers *et al.*, 2023). Only structures with a release date below September 2021 were used for the training, and structures with a release time between September 2021 and January 2023 were for the validation set. The RNA distilled dataset includes predictions from AlphaFold 3 on representative clusters from Rfam (Kalvari *et al.*, 2020) (using a cutoff of 90% of sequence identity and 80% coverage to cluster the sequences into clusters). It leads to an overall of around 65,000 structures for RNAs.

During the training loop, one dataset is sampled, and an example is drawn. Then, a structural crop is made using different strategies: contiguous, spatial, or spatial interface cropping. This strategy was presented in AlphaFold-Multimer (Evans *et al.*, 2021), where the idea is to tackle the high memory and computing required when dealing with long structures. Therefore, the model is trained on cropped segments of full-length molecules, where each subregion is a contiguous block of tokens extracted depending on previously mentioned strategies.

There are four main stages of training for AlphaFold 3. The first uses sequences cropped to 384 tokens, while the rest are considered as fine-tuning. The second stage increases the crop size to 640. The third stage has a crop size of 768 tokens, and the transcription factor distillation sets were made accessible. Finally, the last fine-tuning stage enabled the training of the PAE head and removed the structure-based losses. The initial training took ten days, the second stage 3 days, the third stage took five

days, and the last fine-tuning took two days on 256 NVIDIA A100 GPUs (Abramson *et al.*, 2024).

Differences with AlphaFold 2

AlphaFold last version had to make some changes to its previous version to be adaptable to a large variety of molecules. The different architectures are presented in Figure S1. The first difference is the input, which is not restricted to amino acids. Each residue can be one value among 32: 20 amino acids, 4 RNA nucleotides, 4 DNA nucleotides, a gap (for MSA representation) and three unknown values (one for amino acid, one for RNA nucleotide and one for DNA nucleotide). A second difference is the smaller consideration of the MSA embedding. Instead, this pair representation mainly transmits the information to the diffusion module. The MSA module is only used by the Pairformer module. The third main difference is the removal of the structure module to a diffusion module. This diffusion module does not explicitly consider equivariant processing. Finally, AlphaFold 3 directly outputs the full atom positions, contrary to backbone frames and torsional angles for AlphaFold 2. There are also small differences in the architectures, like the addition of relative token encoding or sequence-local atom attention (where the attention is restricted to a subset of 32 atoms with 128 atoms nearby in sequence space) to prevent huge computation. They also replaced the ReLU activation with SwiGLU, which has proven to be more efficient in their experiments. Each model has a different confidence head: AlphaFold 2 uses experimentally resolved score, pLDDT and pTM for confidence, masked MSA loss, FAPE, structure violation, histogram loss and side chain loss for the final global loss. AlphaFold 3 also uses experimentally resolved score, pLDDT (and pTM) and histogram losses but integrates also the PDE, the diffusion loss and the PAE.

Limitations

AlphaFold 3 has brought novel architecture to enable different molecular structure predictions but remains limited by the diffusion module. Indeed, diffusion-based models tend to have hallucination (Ji *et al.*, 2023). This is reflected by plausible structures in unstructured regions, which did not happen in the previous version of AlphaFold (observed by the authors). Another limitation is the stereochemistry violations: chirality violation and the production of clashing atoms in the predictions. The chirality violation refers to the orientation and symmetry of molecules, where violation could disrupt molecular recognition, binding, and interactions with other molecules. The clashing atoms refer to atoms that are positioned too close to one another, resulting in steric clashes or overlaps. These clashes indicate that the atoms are occupying the same or very close spatial regions, which is energetically unfavourable and physically unrealistic (due to the repulsive forces between the electron clouds of the atoms). The final explicit limitation mentioned by the authors is the non-dynamical predictions, where only static structures are output. A web server is available at https://alphafoldserver.com/ to make online predictions, but it is limited to twenty predictions per day (when writing this article). The source code has been released but requires huge computational resources.

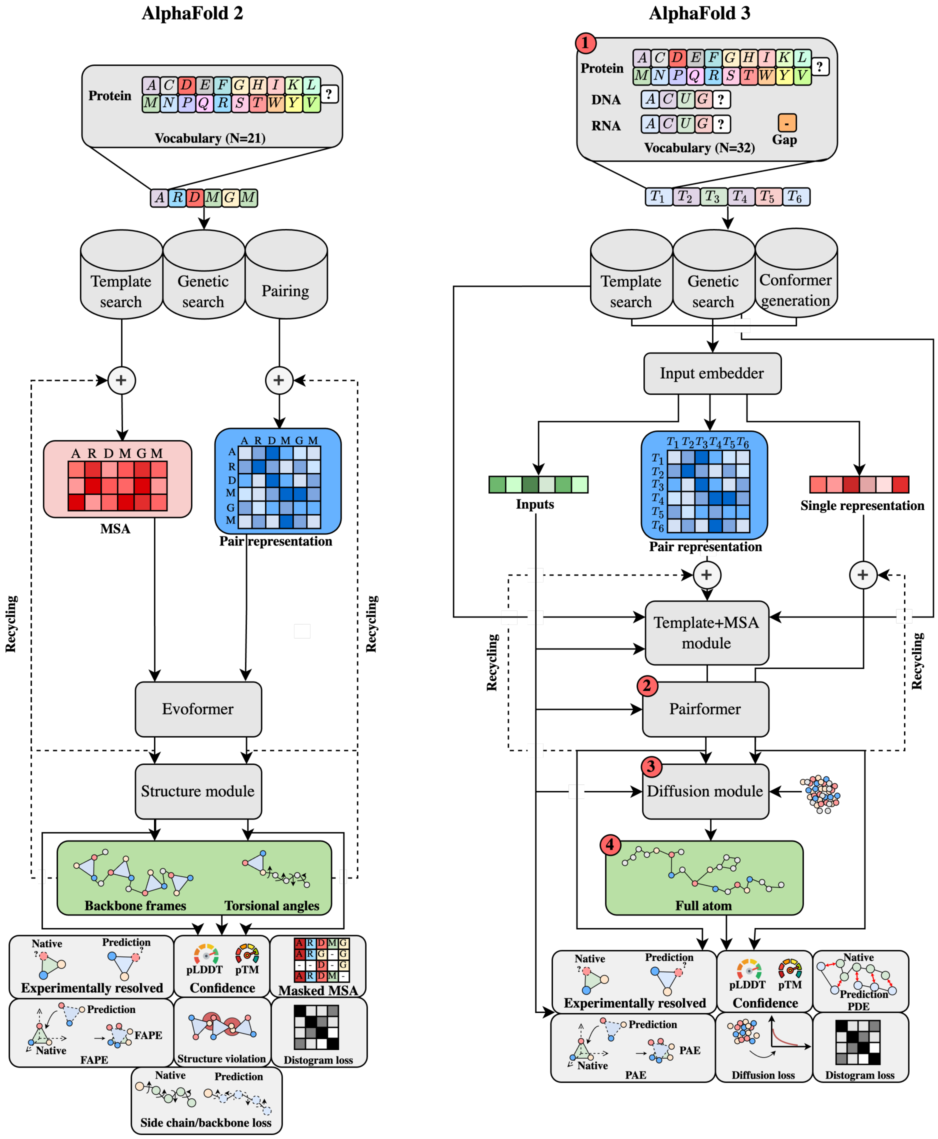

Fig. S1. Architectures of AlphaFold 2 (Jumper *et al.*, 2021) (left) and AlphaFold 3 (Abramson *et al.*, 2024) (right). The key differences are pointed out with red numbers. 1) Difference in terms of inputs: AlphaFold 3 can predict structures of different molecules (proteins, DNAs, RNAs, ligands and ions). 2) The Evoformer is replaced by the Pairformer module, where the MSA is less important, and the pair represents more weights in the network. 3) A diffusion module replaces the structure module to output the final structure. 4) The outputs of the models are different: AlphaFold 3 now outputs directly the positions of atoms, compared to backbone frames and torsional angles for AlphaFold 2.

Results

Table S1. *Mean value (and its standard deviation) for each metric for each predicting model for the RNA-Puzzles dataset.*

Method	RMSD	P-VALUE	ϵRMSD	TM-score	GDT-TS	INF-ALL	CAD	lDDT	MCQ	LCS-10
Challenge-best	**4.94 ± 2.3**	**0.0 ± 0.0**	1.14 ± 0.42	0.42 ± 0.15	0.47 ± 0.21	0.66 ± 0.2	**0.75 ± 0.09**	0.56 ± 0.21	20.77 ± 5.92	15.34 ± 19.26
AlphaFold 3	6.63 ± 6.51	0.01 ± 0.04	**0.88 ± 0.37**	0.57 ± 0.22	0.55 ± 0.3	**0.83 ± 0.12**	0.7 ± 0.28	**0.7 ± 0.14**	**16.18 ± 8.02**	**35.26 ± 31.3**
RNAJP	22.37 ± 10.04	0.19 ± 0.3	1.62 ± 0.37	0.23 ± 0.06	0.25 ± 0.16	0.59 ± 0.16	0.48 ± 0.25	0.06 ± 0.09	25.85 ± 7.07	9.52 ± 6.2
Vfold-Pipeline	16.78 ± 12.56	0.19 ± 0.39	1.42 ± 0.48	0.36 ± 0.18	0.37 ± 0.27	0.69 ± 0.15	0.58 ± 0.27	0.47 ± 0.19	23.82 ± 6.53	13.61 ± 9.43
trRosettaRNA	5.45 ± 5.51	0.0 ± 0.01	1.37 ± 0.22	**0.63 ± 0.22**	**0.59 ± 0.29**	0.64 ± 0.11	0.64 ± 0.29	0.64 ± 0.09	32.05 ± 9.05	6.9 ± 4.51
RhoFold	9.06 ± 6.86	0.01 ± 0.03	1.47 ± 0.59	0.41 ± 0.2	0.4 ± 0.24	0.63 ± 0.18	0.57 ± 0.27	0.51 ± 0.17	52.9 ± 6.96	7.09 ± 21.34
IsRNA1	18.76 ± 9.37	0.24 ± 0.42	1.52 ± 0.28	0.25 ± 0.06	0.28 ± 0.16	0.68 ± 0.11	0.52 ± 0.25	0.43 ± 0.09	24.39 ± 5.5	9.45 ± 5.42
3dRNA	14.24 ± 7.1	0.03 ± 0.14	1.56 ± 0.36	0.31 ± 0.11	0.32 ± 0.21	0.65 ± 0.12	0.53 ± 0.24	0.47 ± 0.14	31.45 ± 7.58	8.82 ± 5.69
SimRNA	29.14 ± 23.8	0.33 ± 0.48	1.68 ± 0.29	0.2 ± 0.11	0.23 ± 0.19	0.49 ± 0.31	0.54 ± 0.2	0.02 ± 0.02	32.08 ± 13.47	5.95 ± 3.87
RNAComposer	20.41 ± 7.35	0.25 ± 0.39	1.5 ± 0.31	0.25 ± 0.06	0.28 ± 0.17	0.66 ± 0.14	0.51 ± 0.26	0.46 ± 0.12	25.36 ± 6.1	10.86 ± 7.22
Vfold3D	18.02 ± 6.76	0.15 ± 0.3	1.77 ± 0.33	0.25 ± 0.06	0.28 ± 0.14	0.56 ± 0.13	0.53 ± 0.22	0.04 ± 0.07	30.3 ± 6.81	4.5 ± nan
MC-Sym	15.56 ± 6.2	0.12 ± 0.3	1.66 ± 0.38	0.26 ± 0.06	0.31 ± 0.17	0.62 ± 0.13	0.54 ± 0.21	0.01 ± 0.01	34.65 ± 2.98	2.08 ± 0.91

Table S2. *Mean value (and its standard deviation) for each metric for each predicting model for the CASP-RNA dataset.*

Method	RMSD	P-VALUE	ϵRMSD	TM-score	GDT-TS	INF-ALL	CAD	lDDT	MCQ	LCS-10
Challenge-best	**9.6 ± 6.29**	**0.0 ± 0.0**	**0.97 ± 0.3**	**0.55 ± 0.16**	**0.4 ± 0.18**	**0.74 ± 0.19**	**0.75 ± 0.24**	**0.63 ± 0.2**	21.46 ± 10.04	17.0 ± 18.68
AlphaFold 3	24.25 ± 13.12	0.0 ± 0.0	1.19 ± 0.28	0.31 ± 0.09	0.27 ± 0.18	0.74 ± 0.15	0.72 ± 0.23	0.59 ± 0.08	**19.3 ± 8.17**	**22.98 ± 27.34**
RNAJP	23.55 ± 6.74	0.23 ± 0.4	1.26 ± 0.19	0.28 ± 0.09	0.26 ± 0.12	0.68 ± 0.15	0.63 ± 0.27	0.04 ± 0.02	25.3 ± 8.48	7.6 ± 4.71
Vfold-Pipeline (TP)	22.92 ± 8.61	0.24 ± 0.34	1.46 ± 0.29	0.28 ± 0.1	0.24 ± 0.1	0.57 ± 0.14	0.6 ± 0.24	0.47 ± 0.08	29.82 ± 8.31	8.38 ± 6.2
trRosettaRNA	22.42 ± 13.79	0.01 ± 0.02	1.59 ± 0.22	0.27 ± 0.11	0.23 ± 0.19	0.52 ± 0.11	0.67 ± 0.21	0.49 ± 0.11	35.88 ± 8.4	4.6 ± 2.95
RhoFold	19.2 ± 7.1	0.0 ± 0.0	2.26 ± 0.44	0.25 ± 0.09	0.24 ± 0.17	0.43 ± 0.16	0.56 ± 0.22	0.2 ± 0.22	61.8 ± 5.24	2.17 ± 1.03
IsRNA1	21.58 ± 7.27	0.08 ± 0.13	1.46 ± 0.29	0.25 ± 0.01	0.24 ± 0.1	0.62 ± 0.15	0.62 ± 0.24	0.42 ± 0.11	25.66 ± 9.86	11.06 ± 7.58
3dRNA	16.28 ± 6.37	0.0 ± 0.0	1.5 ± 0.27	0.29 ± 0.09	0.27 ± 0.14	0.59 ± 0.16	0.61 ± 0.24	0.46 ± 0.1	33.82 ± 7.69	7.98 ± 4.0
SimRNA	24.98 ± 9.91	0.1 ± 0.19	1.62 ± 0.42	0.25 ± 0.04	0.22 ± 0.09	0.61 ± 0.17	0.64 ± 0.24	0.03 ± 0.03	25.1 ± 8.52	7.02 ± 4.43
RNAComposer	29.84 ± 12.87	0.3 ± 0.46	1.35 ± 0.27	0.25 ± 0.09	0.19 ± 0.08	0.65 ± 0.17	0.65 ± 0.22	0.52 ± 0.11	25.98 ± 7.1	8.05 ± 5.57
Vfold3D	22.03 ± 7.61	0.11 ± 0.26	1.58 ± 0.36	0.3 ± 0.1	0.23 ± 0.07	0.54 ± 0.18	0.61 ± 0.24	0.01 ± 0.0	33.24 ± 6.58	nan ± nan
MC-Sym	16.32 ± 2.45	0.4 ± 0.56	2.0 ± nan	0.19 ± 0.03	0.34 ± 0.1	0.5 ± 0.06	0.61 ± 0.07	0.01 ± 0.0	36.53 ± nan	1.45 ± nan

Table S3. *Mean value (and its standard deviation) for each metric for each predicting model for the RNASolo dataset.*

Method	RMSD	P-VALUE	ϵRMSD	TM-score	GDT-TS	INF-ALL	CAD	lDDT	MCQ	LCS-10
AlphaFold 3	6.15 ± 5.95	0.04 ± 0.21	**0.85 ± 0.35**	**0.66 ± 0.2**	0.39 ± 0.28	**0.81 ± 0.15**	**0.4 ± 0.41**	**0.73 ± 0.15**	**15.21 ± 5.59**	**39.41 ± 36.24**
RNAJP	24.84 ± 8.88	0.47 ± 0.45	1.63 ± 0.28	0.26 ± 0.06	0.2 ± 0.11	0.61 ± 0.14	0.31 ± 0.31	0.03 ± 0.03	25.57 ± 3.65	7.81 ± 4.37
Vfold-Pipeline	20.31 ± 11.03	0.22 ± 0.37	1.63 ± 0.42	0.33 ± 0.17	0.19 ± 0.11	0.59 ± 0.18	0.28 ± 0.29	0.42 ± 0.18	25.18 ± 7.12	11.9 ± 9.2
trRosettaRNA	**5.69 ± 7.08**	**0.0 ± 0.0**	1.48 ± 0.25	0.64 ± 0.19	**0.41 ± 0.3**	0.55 ± 0.12	0.38 ± 0.38	0.62 ± 0.1	32.77 ± 5.46	4.78 ± 3.73
RhoFold	10.51 ± 8.93	0.0 ± 0.0	2.12 ± 1.96	0.41 ± 0.18	0.27 ± 0.21	0.53 ± 0.21	0.29 ± 0.32	0.33 ± 0.27	57.97 ± 9.82	2.04 ± 1.02
IsRNA1	24.33 ± 8.77	0.42 ± 0.44	1.69 ± 0.22	0.24 ± 0.05	0.18 ± 0.09	0.6 ± 0.12	0.29 ± 0.3	0.36 ± 0.08	24.49 ± 4.61	7.78 ± 4.55
3dRNA	19.01 ± 8.86	0.09 ± 0.27	1.77 ± 0.3	0.28 ± 0.15	0.19 ± 0.16	0.55 ± 0.15	0.3 ± 0.3	0.36 ± 0.17	33.45 ± 7.52	7.41 ± 5.69
SimRNA	24.11 ± 12.26	0.31 ± 0.41	1.84 ± 0.25	0.25 ± 0.06	0.18 ± 0.1	0.58 ± 0.11	0.31 ± 0.3	0.03 ± 0.02	25.7 ± 3.29	5.07 ± 3.17
RNAComposer	23.47 ± 9.38	0.26 ± 0.36	1.65 ± 0.28	0.24 ± 0.04	0.18 ± 0.1	0.59 ± 0.14	0.32 ± 0.32	0.41 ± 0.12	25.69 ± 5.5	11.57 ± 9.52
Vfold3D	19.54 ± 9.7	0.17 ± 0.34	1.74 ± 0.24	0.3 ± 0.12	0.2 ± 0.1	0.55 ± 0.13	0.3 ± 0.3	0.0 ± 0.0	30.67 ± 3.59	2.91 ± 1.9
MC-Sym	15.89 ± 6.74	0.1 ± 0.2	2.04 ± 0.32	0.24 ± 0.05	0.22 ± 0.08	0.46 ± 0.12	0.31 ± 0.28	0.01 ± 0.02	36.69 ± 4.36	1.92 ± 0.54

Table S4. *Mean value (and its standard deviation) for each metric for each predicting model for the RNA3DB_0 dataset. Results for AlphaFold 3 using context on 113 structures are also provided (AlphaFold 3 (C)).*

Method	RMSD	P-VALUE	ϵRMSD	TM-score	GDT-TS	INF-ALL	CAD	lDDT	MCQ	LCS-10
AlphaFold 3 (C)	**9.08 ± 8.3**	**0.16 ± 0.35**	**0.93 ± 0.5**	**0.44 ± 0.23**	**0.6 ± 0.26**	**0.71 ± 0.24**	**0.78 ± 0.18**	**0.68 ± 0.21**	**22.94 ± 13.39**	**44.28 ± 36.13**
AlphaFold 3	23.19 ± 14.08	0.56 ± 0.48	1.58 ± 0.39	0.25 ± 0.13	0.31 ± 0.19	0.57 ± 0.2	0.67 ± 0.22	0.58 ± 0.15	26.23 ± 11.17	24.1 ± 24.98
RNAJP	23.69 ± 13.15	0.59 ± 0.46	1.58 ± 0.35	0.21 ± 0.08	0.27 ± 0.16	0.5 ± 0.2	0.62 ± 0.2	0.04 ± 0.08	33.1 ± 9.76	9.17 ± 7.91
RhoFold	15.07 ± 12.5	0.34 ± 0.44	1.35 ± 0.52	0.26 ± 0.15	0.35 ± 0.22	0.51 ± 0.23	0.64 ± 0.22	0.53 ± 0.16	63.58 ± 12.76	3.26 ± 2.18
RNAComposer	23.73 ± 13.11	0.57 ± 0.46	1.56 ± 0.36	0.2 ± 0.1	0.27 ± 0.16	0.48 ± 0.2	0.63 ± 0.2	0.51 ± 0.13	33.62 ± 9.83	11.32 ± 13.5

Table S5. *Mean value (and its standard deviation) for each metric for each predicting model for the RNA3DB_Long dataset.*

Method	RMSD	P-VALUE	ϵRMSD	TM-score	GDT-TS	INF-ALL	CAD	lDDT	MCQ	LCS-10
AlphaFold 3	**5.84 ± 6.91**	**0.0 ± 0.0**	**0.66 ± 0.2**	**0.95 ± 0.14**	**0.22 ± 0.24**	**0.87 ± 0.1**	**0.25 ± 0.29**	**0.88 ± 0.13**	**10.92 ± 3.31**	**17.36 ± 18.66**

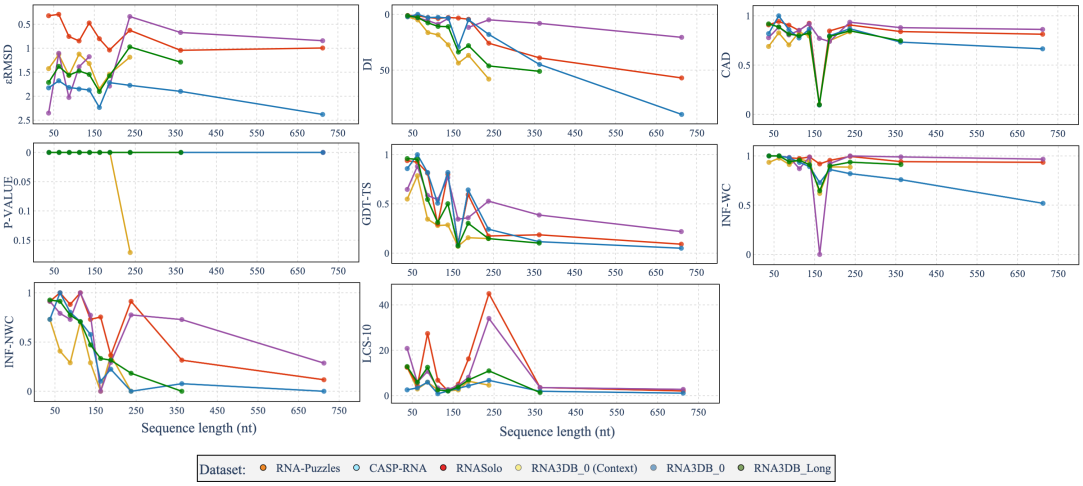

Fig. S2. Averaged metrics depending on the sequence length for the different approaches (AlphaFold 3, *ab initio*, deep-learning, template-based and *challenge-best*). Each point represents the metric averaged over the best models of each approach for a window of 25 nt, from 25 to 750 nt. *Ab initio* methods group RNAJP, IsRNA1 and SimRNA while template-based methods group Vfold-Pipeline, 3dRNA, RNAComposer, Vfold3D and MC-Sym. Deep learning methods group trRosettaRNA and RhoFold. Metrics are computed for the RNA-Puzzles, CASP-RNA and RNASolo datasets. The *Challenge-best* corresponds to the best results from either RNA-Puzzles or CASP-RNA competitions but does not appear for the RNASolo dataset. The metrics are ϵRMSD, DI, CAD-score, P-VALUE, GDT-TS, INF-WC, INF-NWC and LCS-10. ϵRMSD, and DI are reversed to have the best values near the top and the worst values at the bottom.

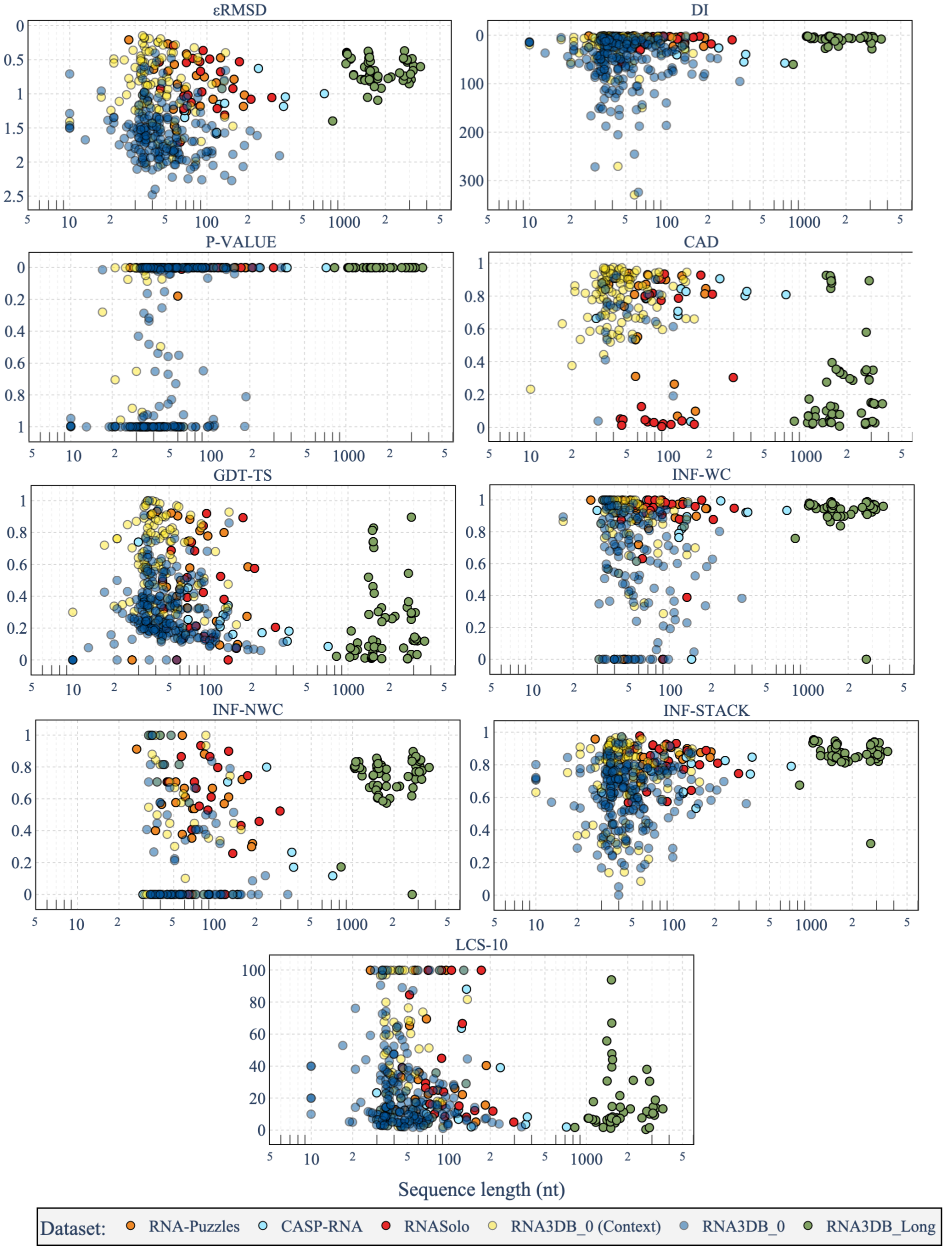

Fig. S3. Dependence of metrics with the sequence length on the prediction of AlphaFold 3 (Abramson *et al.*, 2024) on the five test sets. The metrics are ϵRMSD, DI, CAD-score, P-VALUE, GDT-TS, INF-WC, INF-NWC and LCS-10. ϵRMSD and DI are reversed to have the best values near the top and the worst values at the bottom.

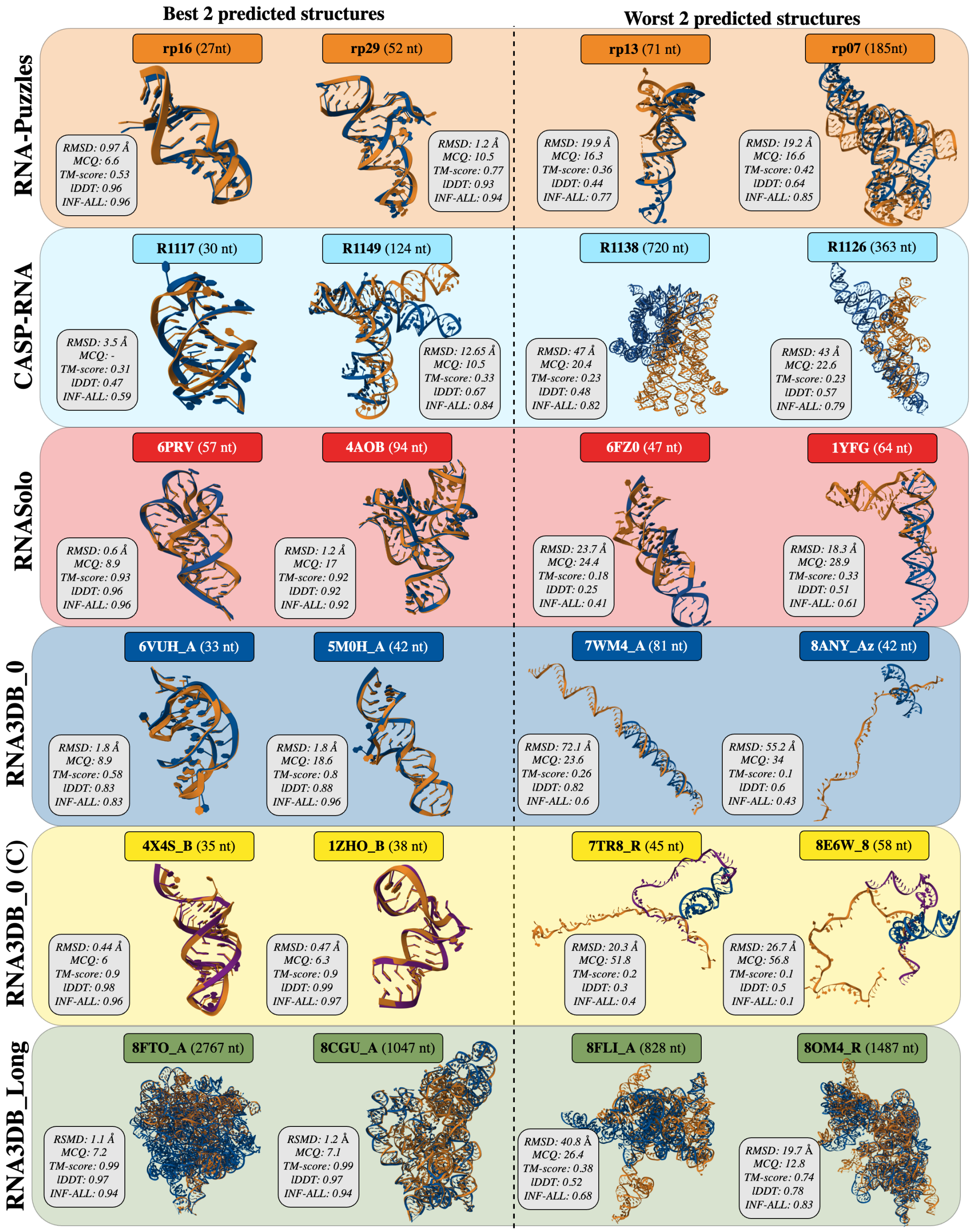

Fig. S4. The two best and two worst predictions from AlphaFold 3 for RNA-Puzzles, CASP-RNA RNASolo, RNA3DB_0 and RNA3DB_Long based on cumulative normalised metrics. The RMSD, MCQ, TM-score, lDDT and INF-ALL are included for each structure. The predictions from AlphaFold 3 (in blue) are aligned with the native ones (in orange) using US-align (Zhang *et al.*, 2022*a*). Predictions from AlphaFold 3 with context for RNA3DB are also provided (in purple).

Table S6. *INF (Parisien* et al., *2009) metrics (INF-WC, INF-NWC and INF-STACK) for each method for three datasets: CASP-RNA, RNA-Puzzles and RNASolo. The Challenge-best corresponds to the best results from either RNA-Puzzles or CASP-RNA competitions. Methods are AlphaFold 3 (Abramson* et al., *2024), RNAJP (Jun & Shi-Jie, 2023), VFold-Pipeline (Li* et al., *2022), trRosettaRNA (Wang* et al., *2023), RhoFold (Shen* et al., *2022), IsRNA1 (Zhang* et al., *2021), 3dRNA (Zhang* et al., *2022*b*), SimRNA (Boniecki* et al., *2016), RNAComposer (Popenda* et al., *2012), Vfold3D (Cao & Chen, 2011) and MC-Sym (Parisien & Major, 2008). Methods are sorted by release time (except for challenge-best.)*

	CASP-RNA			RNA-Puzzles			RNASolo		
	WC	NWC	STACK	WC	NWC	STACK	WC	NWC	STACK
Challenge-best	**0.81**	**0.38**	0.73	0.60	0.22	0.64	-	-	-
AlphaFold 3	**0.81**	0.21	**0.74**	**0.89**	**0.55**	**0.83**	**0.83**	**0.51**	**0.82**
RNAJP	0.69	0.12	0.72	0.55	0.15	0.65	0.59	0.19	0.67
Vfold-Pipeline	0.63	0.00	0.56	0.78	0.29	0.70	0.62	0.14	0.62
trRosettaRNA	0.50	0.03	0.54	0.60	0.13	0.57	0.53	0.07	0.52
RhoFold	0.31	0.03	0.54	0.70	0.20	0.66	0.50	0.10	0.60
IsRNA1	0.65	0.00	0.65	0.80	0.00	0.68	0.61	0.01	0.64
3dRNA	0.61	0.02	0.61	0.73	0.17	0.64	0.57	0.07	0.56
SimRNA	0.59	0.10	0.65	0.68	0.06	0.59	0.57	0.08	0.65
RNAComposer	0.73	0.10	0.65	0.75	0.33	0.67	0.62	0.21	0.62
Vfold3D	0.57	0.02	0.58	0.73	0.13	0.65	0.61	0.06	0.59
MC-Sym	0.45	0.00	0.58	0.68	0.04	0.64	0.26	0.12	0.54

Table S7: RMSD, MCQ, TM-Score, INF and lDDT for the RNA3DB_0 dataset (only the structures predicted by AlphaFold 3 with and without context) for the predictions of AlphaFold 3 (with context on the left (C) and without context on the right (wC)).

**RNA name**	**RMSD**	**MCQ**	**TM-Score**	**INF-ALL**	**lDDT**
	C/wC	C/wC	C/wC	C/wC	C/wC
1G1X_D	0.61/19.01	8.47/14.74	0.87/0.26	0.94/0.74	0.86/0.61
1GTN_W	30.80/30.60	44.56/45.94	0.08/0.07	0.11/0.43	0.31/0.24
1KUQ_B	0.90/4.77	15.36/19.85	0.89/0.44	0.95/0.81	0.81/0.69
1S03_B	2.36/4.67	18.24/19.85	0.72/0.29	0.92/0.77	0.82/0.65
1YKV_B	1.05/6.05	15.22/17.34	0.66/0.27	0.90/0.77	0.87/0.62
1ZHO_B	0.48/4.69	6.33/14.92	0.90/0.31	0.97/0.95	0.99/0.71
3CIY_C	2.40/32.63	8.08/13.11	0.55/0.21	0.93/0.67	0.95/0.69
3EGZ_B	3.31/6.84	11.72/13.62	0.60/0.37	0.83/0.78	0.72/0.67
3HHN_C	1.04/3.26	10.16/12.17	0.96/0.90	0.91/0.90	0.95/0.80
3ID5_D	4.91/17.15		0.54/0.16	0.95/0.47	0.81/0.35

Continued on next page

Table S7 – continued from previous page

RNA	RMSD	MCQ	TM-Score	INF-ALL	lDDT
	C/wC	C/wC	C/wC	C/wC	C/wC
3NMU_D	0.72/13.45	12.25/26.52	0.81/0.21	0.93/0.73	0.96/0.55
4B3P_R	10.57/14.51	9.31/10.19	0.42/0.19	0.87/0.67	0.78/0.70
4B3Q_R	3.66/19.09	9.94/12.02	0.34/0.17	0.91/0.65	0.85/0.66
4C7O_E	1.15/5.60	9.92/16.26	0.70/0.41	0.96/0.90	0.93/0.77
4K27_U	2.17/2.60	9.76/10.29	0.64/0.58	0.92/0.93	0.87/0.87
4K50_B	1.23/8.65		0.54/0.28	0.93/0.89	0.99/0.89
4KR9_M	3.54/7.12	25.38/27.04	0.61/0.35	0.85/0.74	0.76/0.58
4M4O_B	0.77/8.23	7.49/16.39	0.89/0.45	0.95/0.75	0.96/0.63
4MGN_A	1.51/7.64	8.67/9.99	0.83/0.43	0.93/0.85	0.86/0.69
4NI7_B			0.12/0.14	0.19/0.25	0.18/0.34
4OOG_D	0.83/3.47	8.80/12.52	0.66/0.41	0.94/0.92	0.97/0.84
4PMI_A	2.40/2.61	10.97/12.64	0.50/0.43	0.92/0.93	0.86/0.84
4RMO_P	2.73/5.77	11.61/18.98	0.59/0.30	0.87/0.74	0.85/0.58
4X4S_B	0.44/3.10	6.01/12.02	0.91/0.38	0.96/0.90	0.98/0.83
5BTM_B	9.11/2.69	14.32/13.58	0.53/0.48	0.82/0.93	0.70/0.89
5DCV_B	2.56/6.03	27.10/29.36	0.58/0.41	0.83/0.82	0.80/0.81
5DEA_A	2.52/6.42	24.80/27.24	0.46/0.29	0.86/0.49	0.69/0.37
5FJ4_D	0.44/4.84	7.53/16.82	0.92/0.34	0.96/0.86	0.99/0.77
5GIN_G	29.89/33.83	10.23/23.09	0.40/0.18	0.69/0.65	0.92/0.62
5HRT_B	5.58	26.01	0.19/0.17	0.86/0.29	0.57/0.22
5M0H_A	2.10/1.82	15.61/18.53	0.60/0.80	0.96/0.96	0.84/0.88
5VOE_A	2.12/5.02	18.24/17.54	0.50/0.30	0.84/0.78	0.43/0.39
5WLH_B	0.90/13.59		0.84/0.26	0.95/0.64	0.94/0.50
5XWY_B	10.77/20.17	23.16/32.58	0.50/0.20	0.73/0.49	0.70/0.47
5Y7M_B	3.71/13.58	12.37/17.91	0.57/0.28	0.86/0.74	0.83/0.62
6AAY_B	3.81/16.51	22.75/24.09	0.57/0.18	0.81/0.63	0.77/0.62
6E82_A	12.65/13.35	32.98/34.28	0.26/0.26	0.55/0.54	0.39/0.37
6E9E_B	2.77/21.30	12.95/24.14	0.67/0.30	0.66/0.41	0.82/0.52
6IFR_J	10.66/39.25	23.33/29.40	0.36/0.11	0.86/0.56	0.77/0.65
6IFU_I	10.81/38.78	15.96/28.39	0.42/0.14	0.74/0.46	0.81/0.61
6IV8_D	2.88/16.72	15.31/24.74	0.69/0.32	0.88/0.65	0.84/0.59
6LSH_B	10.36/8.34	17.33/30.59	0.32/0.29	0.54/0.59	0.45/0.69
6LTP_B	12.87/24.21	25.09/33.41	0.53/0.16	0.80/0.45	0.81/0.56
6O1O_M	6.87/37.89	37.57/36.32	0.17/0.15	0.67/0.44	0.55/0.54
6O1O_N	8.41/36.91	32.76/37.08	0.21/0.14	0.65/0.45	0.72/0.62
6SY4_C	1.39/14.67		0.64/0.28	0.89/0.65	0.89/0.51
6VUH_A	1.13/1.81	9.74/8.88	0.65/0.58	0.84/0.83	0.87/0.83
6WPI_B	4.36/8.65		0.73/0.59	0.89/0.86	0.91/0.84
6WW6_D	6.70/3.75	18.95/12.73	0.34/0.26	0.78/0.83	0.61/0.71
6XJY_B	11.86/11.33	20.48/18.82	0.24/0.31	0.61/0.64	0.48/0.51
6XMF_C	3.62/24.12	15.29/19.50	0.73/0.29	0.87/0.73	0.77/0.52
6XN4_R	10.27/35.50	36.33/42.78	0.18/0.13	0.35/0.27	0.61/0.51

Continued on next page

Table S7 – continued from previous page

RNA	RMSD	MCQ	TM-Score	INF-ALL	lDDT
	C/wC	C/wC	C/wC	C/wC	C/wC
7CYQ_I	5.52/21.17	8.65/16.84	0.47/0.20	0.90/0.68	0.82/0.66
7CYQ_J	18.87/17.65	14.68/13.72	0.38/0.29	0.77/0.72	0.62/0.78
7D8O_L	4.75/14.57		0.51/0.31	0.87/0.39	0.82/0.38
7DLZ_X	2.01/6.56	19.55/24.62	0.69/0.28	0.85/0.72	0.82/0.57
7DMQ_B	4.61/32.08	16.13/21.91	0.44/0.19	0.78/0.50	0.77/0.60
7DMQ_C	2.09/23.10	17.94/19.44	0.38/0.27	0.77/0.60	0.86/0.79
7DTE_F	7.49/29.45	12.39/11.78	0.39/0.26	0.82/0.60	0.82/0.80
7DTE_G	3.14/25.05	9.10/9.30	0.46/0.26	0.83/0.75	0.88/0.86
7EU0_O	5.00/37.07	47.62/39.70	0.22/0.17	0.84/0.50	0.51/0.60
7JL2_X	3.97/40.40	6.65/9.27	0.29/0.23	0.89/0.60	0.91/0.81
7JL2_Y	3.93/42.44	9.14/11.24	0.36/0.21	0.84/0.61	0.93/0.84
7JNH_B	1.31/5.49	15.39/16.27	0.86/0.48	0.89/0.81	0.91/0.75
7JRS_B	17.95/19.62	9.99/9.86	0.37/0.34	0.85/0.86	0.79/0.79
7JRT_B	16.24/16.32	14.91/15.07	0.36/0.32	0.85/0.87	0.72/0.74
7KHA_J	17.76/44.24	31.19/43.34	0.29/0.13	0.41/0.29	0.68/0.55
7KVU_G	10.45/11.44	18.50/18.25	0.31/0.28	0.56/0.54	0.47/0.45
7KWG_A	7.67/21.30	41.23/39.82	0.20/0.12	0.38/0.50	0.51/0.56
7L49_E	21.59/31.02	18.15/18.37	0.56/0.26	0.72/0.70	0.59/0.54
7M5O_B	6.27/15.34	24.65/30.17	0.52/0.34	0.78/0.63	0.81/0.70
7OAW_B	11.20/11.31	16.49/15.96	0.33/0.34	0.60/0.64	0.50/0.51
7OS0_D	8.39/14.71		0.40/0.18	0.68/0.51	0.61/0.48
7OZQ_E	8.80/8.99	10.22/11.65	0.41/0.35	0.80/0.77	0.74/0.71
7R6L_B	13.55/21.11	33.42/33.71	0.19/0.15	0.38/0.37	0.40/0.55
7SBA_Z	11.04/46.49	38.34/30.20	0.14/0.14	0.36/0.39	0.45/0.55
7SBB_X	7.77/37.63	45.71/40.56	0.17/0.13	0.75/0.59	0.62/0.65
7TQV_G	4.91/31.02	12.27/19.19	0.25/0.23	0.88/0.64	0.81/0.66
7TQV_H	16.53/29.44	11.15/8.54	0.24/0.19	0.78/0.73	0.62/0.80
7TR8_R	25.51/40.91	43.52/36.77	0.14/0.10	0.28/0.36	0.40/0.48
7V00_G	13.81/36.94	42.79/46.24	0.36/0.12	0.30/0.14	0.66/0.54
7V94_B	9.72/20.78	17.49/17.64	0.52/0.32	0.77/0.57	0.57/0.43
7VG2_C	5.54/23.27	12.25/16.58	0.28/0.24	0.95/0.69	0.91/0.75
7VG2_D	6.06/27.22	9.42/11.60	0.23/0.25	0.90/0.73	0.86/0.82
7VTI_B	6.45/10.50	26.11/20.16	0.28/0.21	0.76/0.69	0.54/0.56
7VYX_D	21.57/23.48	34.39/30.24	0.27/0.25	0.56/0.56	0.40/0.45
7WM4_A	5.72/72.12	22.89/23.57	0.39/0.27	0.74/0.60	0.84/0.82
7X7R_J	20.35/15.37	51.80/43.44	0.17/0.15	0.36/0.26	0.34/0.58
7YNB_C	14.54/30.98	33.32/30.37	0.12/0.12	0.68/0.49	0.65/0.68
7YNC_B	8.05/39.15	40.42/36.48	0.31/0.12	0.31/0.33	0.47/0.55
7ZOQ_C	23.02/40.60	44.62/42.42	0.23/0.15	0.25/0.26	0.44/0.52
8ANE_R	36.85/42.37	42.17/42.68	0.13/0.12	0.25/0.30	0.54/0.52
8BVJ_B	27.55/25.58	56.02/47.18	0.14/0.26	0.08/0.42	0.21/0.55
8D1V_J	7.39/34.26	44.65/43.69	0.46/0.16	0.13/0.23	0.44/0.49

Continued on next page

Table S7 – continued from previous page

RNA	RMSD	MCQ	TM-Score	INF-ALL	lDDT
	C/wC	C/wC	C/wC	C/wC	C/wC
8D29_R	5.78/6.07	17.74/19.47	0.29/0.27	0.75/0.79	0.77/0.73
8DO6_I	11.82/36.89	31.33/27.87	0.42/0.14	0.60/0.53	0.56/0.48
8DO6_J	9.04/28.89	32.99/27.01	0.24/0.12	0.43/0.59	0.50/0.70
8E6W_8	26.62/35.38	56.86/58.10	0.12/0.10	0.08/0.14	0.50/0.50
8EWG_B	16.03/24.98	40.44/39.92	0.19/0.20	0.28/0.21	0.47/0.36
8F0N_B	12.00/13.37	26.96/30.22	0.29/0.28	0.59/0.58	0.44/0.47
8FF5_M	34.22/54.28	40.44/40.18	0.19/0.10	0.38/0.29	0.52/0.48
8FN6_G	15.42/8.56	23.28/16.70	0.37/0.48	0.35	0.37/0.63
8GH6_R	28.63/15.46	32.54/23.03	0.12/0.23	0.21/0.55	0.07/0.52
8GKH_W	6.94/32.06	26.09/22.23	0.43/0.31	0.75/0.44	0.63/0.39
8GXB_B	8.51/9.07	17.63/20.40	0.34/0.31	0.69/0.67	0.56/0.53
8H7Q_D	10.41/41.79	51.83/45.84	0.21/0.13	0.43/0.38	0.53/0.52
8HB8_A	11.87/13.77	22.12/23.67	0.28/0.26	0.59/0.48	0.36/0.33
8HSR_R	24.46/23.77	59.80/29.84	0.12/0.22	0.50/0.54	0.19/0.73
8HUD_B	1.56/14.70	14.18/17.71	0.86/0.42	0.81/0.73	0.88/0.74
8IBW_D	10.56/11.65	31.35/26.22	0.31/0.26	0.71/0.69	0.56/0.59
8J12_C	25.45/34.73	19.33/20.91	0.33/0.25	0.69/0.59	0.49/0.45
8T29_R	7.92/13.04	23.12/23.54	0.52/0.49	0.76/0.77	0.63/0.53
8UPT_A	3.69/3.76	31.25/31.87	0.68/0.60	0.81/0.78	0.72/0.67

References

Abramson, J., Adler, J., Dunger, J., Evans, R., Green, T., Pritzel, A., Ronneberger, O., Willmore, L., Ballard, A. J., Bambrick, J., Bodenstein, S. W., Evans, D. A., Hung, C.-C., O'Neill, M., Reiman, D., Tunyasuvunakool, K., Wu, Z., Žemgulytė, A., Arvaniti, E., Beattie, C., Bertolli, O., Bridgland, A., Cherepanov, A., Congreve, M., Cowen-Rivers, A. I., Cowie, A., Figurnov, M., Fuchs, F. B., Gladman, H., Jain, R., Khan, Y. A., Low, C. M. R., Perlin, K., Potapenko, A., Savy, P., Singh, S., Stecula, A., Thillaisundaram, A., Tong, C., Yakneen, S., Zhong, E. D., Zielinski, M., Žídek, A., Bapst, V., Kohli, P., Jaderberg, M. & Hassabis, D. (2024). *Nature*, **630**(493), 493–500.

Boniecki, M. J., Lach, G., Dawson, W. K., Tomala, K., Lukasz, P., Soltysinski, T., Rother, K. M. & Bujnicki, J. M. (2016). *Nucleic Acids Research*, **44**, e63–e63.

Cao, S. & Chen, S.-J. (2011). *Journal of Physical Chemistry B*, **115**, 4216–4226.

Consortium, R. (2021). *Nucleic Acids Research*, **49**(D1), D212–D220.

Evans, R., O'Neill, M., Pritzel, A., Antropova, N., Senior, A., Green, T., Žídek, A., Bates, R., Blackwell, S., Yim, J., Ronneberger, O., Bodenstein, S., Zielinski, M., Bridgland, A., Potapenko, A., Cowie, A., Tunyasuvunakool, K., Jain, R., Clancy, E., Kohli, P., Jumper, J. & Hassabis, D. (2021). *bioRxiv*, p. 2021.10.04.463034. Preprint.

Ji, Z., Lee, N., Frieske, R., Yu, T., Su, D., Xu, Y., Ishii, E., Bang, Y. J., Madotto, A. & Fung, P. (2023). *ACM Comput. Surv.* **55**(12).

Jumper, J., Evans, R., Pritzel, A., Green, T., Figurnov, M., Ronneberger, O., Tunyasuvunakool, K., Bates, R., Žídek, A., Potapenko, A., Bridgland, A., Meyer, C., Kohl, S. A. A., Ballard, A. J., Cowie, A., Romera-Paredes, B., Nikolov, S., Jain, R., Adler, J., Back, T., Petersen, S., Reiman, D., Clancy, E., Zielinski, M., Steinegger, M., Pacholska, M., Berghammer, T., Bodenstein, S., Silver, D., Vinyals, O., Senior, A. W., Kavukcuoglu, K., Kohli, P. & Hassabis, D. (2021). *Nature*, **596**, 583–589.

Jun, L. & Shi-Jie, C. (2023). *Nucleic Acids Research*, **51**, 3341–3356.

Kagaya, Y., Zhang, Z., Ibtehaz, N., Wang, X., Nakamura, T., Huang, D. & Kihara, D. (2023). *bioRxiv.*

Kalvari, I., Nawrocki, E. P., Ontiveros-Palacios, N., Argasinska, J., Lamkiewicz, K., Marz, M., Griffiths-Jones, S., Toffano-Nioche, C., Gautheret, D., Weinberg, Z., Rivas, E., Eddy, S. R., Finn, R. D., Bateman, A. & Petrov, A. I. (2020). *Nucleic Acids Research.*

Li, J., Zhang, S., Zhang, D. & Chen, S.-J. (2022). *Bioinformatics*, **38**(16), 4042–4043.

Li, Y., Zhang, C., Feng, C., Pearce, R., Freddolino, P. L. & Zhang, Y. (2023). *Nature Communications*, **14**, 5745.

Parisien, M., Cruz, J., Westhof, E. & Major, F. (2009). *RNA (New York, N.Y.)*, **15**, 1875–85.

Parisien, M. & Major, F. (2008). *Nature*, **452**, 51–55.

Popenda, M., Szachniuk, M., Antczak, M., Purzycka, K. J., Lukasiak, P., Bartol, N., Blazewicz, J. & Adamiak, R. W. (2012). *Nucleic Acids Research*, **40**, e112–e112.

Sayers, E. W., Bolton, E. E., Brister, J. R., Canese, K., Chan, J., Comeau, D. C., Farrell, C. M., Feldgarden, M., Fine, A. M., Funk, K., Hatcher, E., Kannan, S., Kelly, C., Kim, S., Klimke, W., Landrum, M. J., Lathrop, S., Lu, Z., Madden, T. L., Malheiro, A., Marchler-Bauer, A., Murphy, T. D., Phan, L., Pujar, S., Rangwala, S. H., Schneider, V. A., Tse, T., Wang, J., Ye, J., Trawick, B. W., Pruitt, K. D. & Sherry, S. T. (2023). *Nucleic acids research*, **51**(D1), D29–D38.

Sha, C. M., Wang, J. & Dokholyan, N. V. (2023). *Biophysical Journal*, **122**(3, Supplement 1), 444a.

Shen, T., Hu, Z., Peng, Z., Chen, J., Xiong, P., Hong, L., Zheng, L., Wang, Y., King, I., Wang, S., Sun, S. & Li, Y. (2022). *arXiv preprint arXiv:2207.01586.*

Wang, W., Feng, C., Han, R., Wang, Z., Ye, L., Du, Z., Wei, H., Zhang, F., Peng, Z. & Yang, J. (2023). *Nat Commun*, **14**, 7266.

Zhang, C., Shine, M., Pyle, A. M. & Zhang, Y. (2022*a*). *Nature Methods*, **19**, 1109–1115.

Zhang, D., Li, J. & Chen, S.-J. (2021). *Journal of Chemical Theory and Computation*, **17**, 1842–1857.

Zhang, Y., Wang, J. & Xiao, Y. (2022*b*). *Journal of Molecular Biology*, **434**(11), 167452. Computation Resources for Molecular Biology.
